# Mesomeric tuning of merocyanine photoacids

**DOI:** 10.1039/d6sc06885e

**Published:** 2026-10-26

**Authors:** Bharti Yadav, Saif Eddine Cherif, Stefano Contena, Gianpiero Cera, Marzio Rancan, Barbara Fresch, Jack Li-Yang Chen, Cristian Pezzato

**Affiliations:** a Laboratory for Macromolecular and Organic Chemistry, Department of Chemical Sciences, University of Padova Via Marzolo 1 35131 Padova Italy cristian.pezzato@unipd.it; b Department of Biotechnology, Chemistry and Pharmaceutical Sciences, Università Degli Studi di Siena 53100 Siena Italy; c Dipartimento di Scienze Chimiche, Della Vita e Della Sostenibilità Ambientale, Università di Parma Parco Area Delle Scienze 17/A I-43124 Parma Italy; d Institute of Condensed Matter Chemistry and Technologies for Energy (ICMATE), National Research Council (CNR), c/o Department of Chemistry, University of Padova Via F. Marzolo 1 35131 Padova Italy; e Department of Chemical Sciences, University of Padova Via Marzolo 1 35131 Padova Italy; f School of Science, Auckland University of Technology 34 St Paul St Auckland 1142 New Zealand; g The MacDiarmid Institute for Advanced Materials and Nanotechnology Wellington New Zealand

## Abstract

We synthesized a library of methoxy-substituted merocyanine photoacids to systematically investigate how mesomeric effects influence their hydrolytic stability and photoacid properties. Our results demonstrate that multiple methoxy groups directly conjugated to the ene-iminium core exert a cumulative electron-donating effect, with the bis-substituted derivative 4 exhibiting a nearly three-fold longer half-life than either of the mono-substituted analogues 2 or 3 (3.5 *vs.* 1.3 days), which in turn are nearly three times more hydrolytically stable than unsubstituted compound 1 (1.3 *vs.* 0.5 days). Compound 4 displays an acidity constant close to neutrality (p*K*_a_^GS^ = 7.1), which decreases by more than 3 p*K* units (p*K*_a_^MS^ = 3.9) upon 500 nm light irradiation, as determined by UV-Vis spectrophotometric titrations under dark conditions and in the photostationary state, respectively. These properties enabled the preparation of visible light-switchable buffer solutions featuring basic-to-acidic pH jumps of 3.2 units that can be reversibly triggered over at least two consecutive days with minimal loss, highlighting the potential of MCH 4 in solar energy harvesting applications.

## Abstract 2

Cumulative electron donation from multiple conjugated methoxy groups protects the ene-iminium core against hydrolysis while preserving merocyanine photoacid properties.

## Graphical abstract

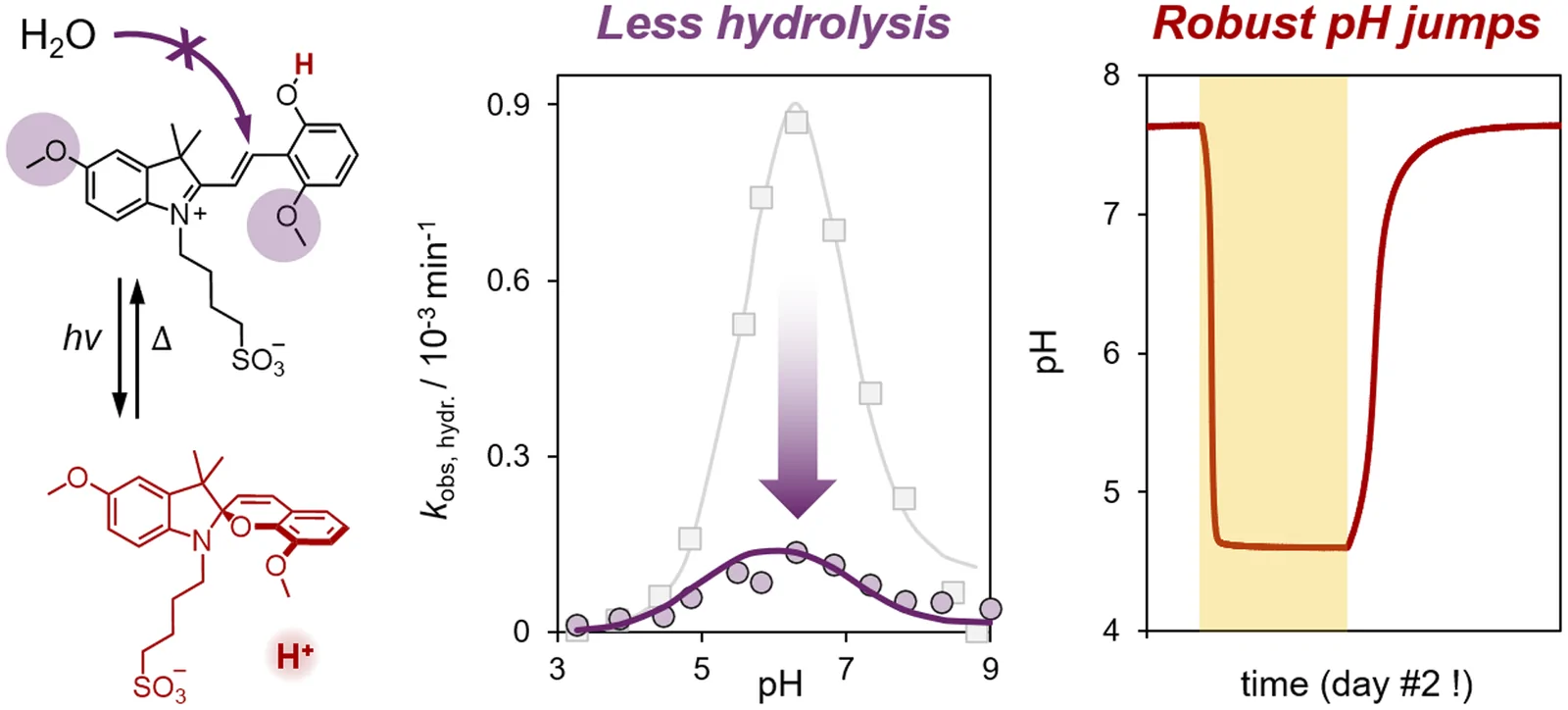

## Introduction

Metastable-state photoacids (mPAHs), which can reversibly generate metastable proton gradients under moderate irradiation, have become a common tool for driving and controlling aqueous-based chemical processes with light.^1–5^ Upon visible light irradiation, these molecules photoisomerize to long-lived, strongly acidic states that can thermally revert to their original state in the dark. The ability to reversibly modulate pH with high spatial and temporal precision has unlocked numerous applications across diverse research areas including chemistry,^6–9^ materials science,^10–15^ biology^7,16^ and energy technology.^4,17–19^

Among mPAHs, merocyanines (MCHs) are the most extensively studied photochromic switches.^1,3,4,17,18,20,21^ Their photoacidity (*Π*) arises from thermally and photochemically interconvertible species, which include the open protonated (*trans*-MCH and *cis*-MCH) and deprotonated (MC) merocyanines, as well as the respective closed spiropyran (SP) isomer (Fig. 1a). Typically, a p*K*_a_ shift from approximately 6–7 in the ground state (p*K*_a_^GS^) to as low as 2–3 in the metastable state (p*K*_a_^MS^) is observed – *i.e.*, *Π* = p*K*_a_^GS^ − p*K*_a_^MS^ ≈ 4. Under dark conditions, the system relaxes back to the thermodynamically most stable open merocyanine state, restoring its initial acidity.^2,4,15,22–25^ This reversible, state-dependent acidity change underpins the exceptional versatility of MCH photoacids and has enabled emerging applications in DNA nanotechnology, including the dynamic regulation of phase-separated microdroplets^26^ and construction of supramolecular architectures.^27–29^ Beyond biomolecular systems, MCH photoacids have also been exploited for reversible CO_2_ capture and release^30–34^ where light-induced proton transfer regulates carbonate/bicarbonate equilibria, as well as in devices for light-to-protonic energy conversion.^4,17^

**Fig. 1:**
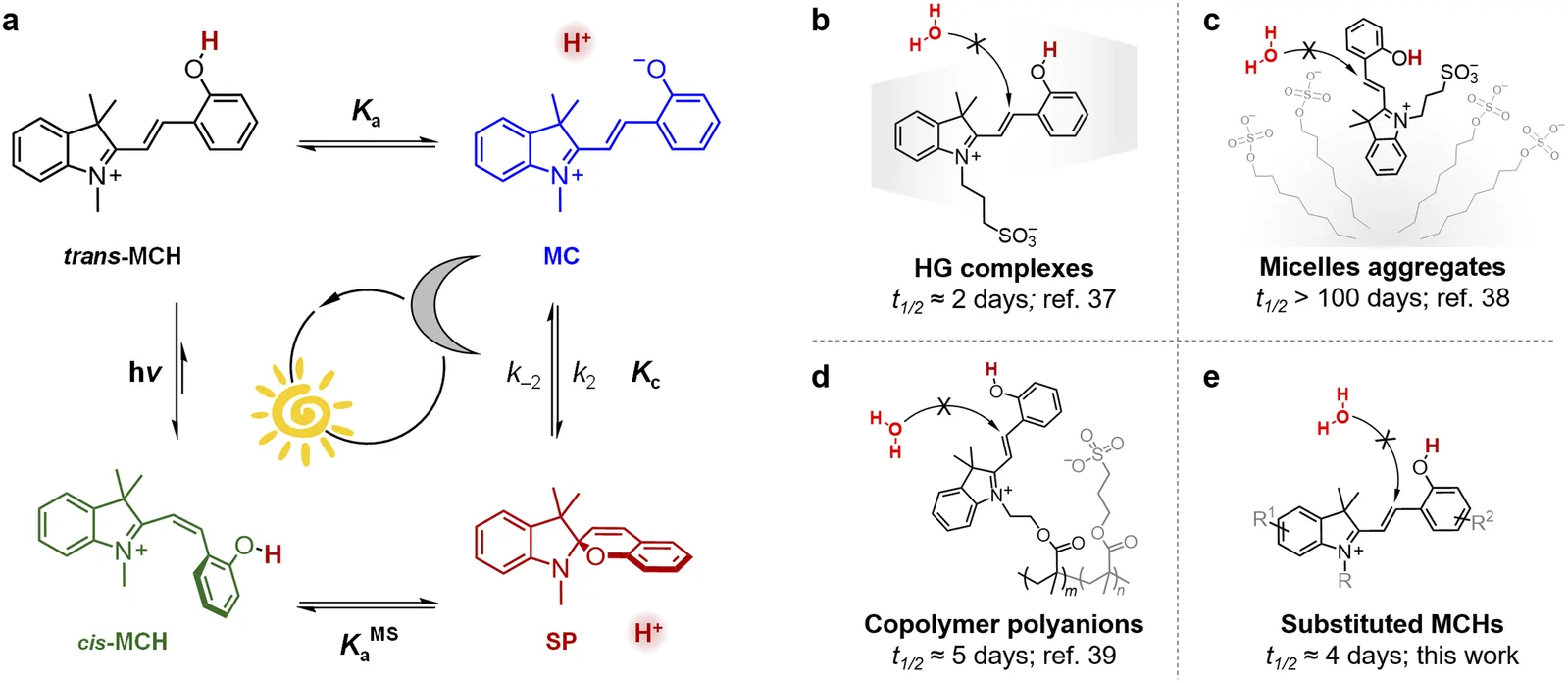
(a) Four-state model illustrating the reversible photoacid behaviour of MCHs, which involves reversible reactions between *trans*-MCH, *cis*-MCH, SP, and MC isomers. Strategies for enhancing the hydrolytic stability of MCHs include (b) host–guest (HG) complex formation,^37^ (c) intercalation into micellar aggregates,^38^ (d) incorporation into copolymer polyanions,^39^ and (e) a substitution-based molecular strategy (this work).

These emerging applications highlight the tremendous potential of MCH-based photoacids. However, their real-world implementation remains significantly limited by several critical factors, the most relevant of which is hydrolytic stability.^3^ Recent studies indicate that, while *Π* typically spans 3–4 p*K* units, MCHs readily undergo hydrolysis, and their hydrolytic stability is strongly influenced by subtle molecular modifications.^25,35,36^ These factors directly impact the magnitude, reversibility, and longevity of the generated proton gradients, thereby restricting their applicability. The development of reversible photoacids with enhanced (photo)chemical stability in aqueous environments is thus essential for developing robust light-controlled pH-switching systems.^17,25^

Various strategies have been developed to improve the hydrolytic stability of metastable photoacids (MCHs), including host–guest (HG) inclusion complexes,^37^ incorporation into SDS micellar aggregates,^38^ and copolymer polyanion systems^39^ (Fig. 1b–d, respectively). More recently, molecular substitution of the parent MCH scaffold^23^ (R^1^ = H, R^2^ = H, R = (CH_2_)_3_SO_3_^−^) has emerged as an effective strategy to improve photoacid performance.^17,25,35,36,40–42^ Seminal work by Liao and co-workers^40^ indicates that *para*-methoxy substitution on the chromene moiety improves hydrolytic stability as compared to the parent MCH scaffold.^23^ Consistent with this trend, our group^35^ showed that introducing a *para*-methoxy group either on the chromene or the indolinium moiety extends the half-life in water. However, the former derivative was found to be poorly photoactive.^35^ In parallel, both Beves'^42^ and our group^25^ demonstrated that adding a butyl-1-sulfonate spacer enhances the hydrolytic stability compared to that of propyl-1-sulfonate analogues.^35^ Building upon these findings, this work (Fig. 1e) systematically investigates cumulative effects by synthesizing analogues of the butyl-1-sulfonate scaffold (R = (CH_2_)_4_SO_3_^−^) with methoxy groups on the indolinium (R^1^ = *p*-OMe) and/or the chromene (R^2^ = *o*-OMe) moieties, and establishes quantitative structure–property relationships that govern both hydrolytic stability and photoacid properties in water.

## Results and discussion

A library of four different merocyanines was synthesized according to established literature procedures (Scheme 1).^25,36,42^ Unsubstituted compound 1 was taken as the reference for comparative evaluations.^25^ Precursor I1 is commercially available. In the first step, compound I2 was obtained through a Fischer indole reaction of 4-methoxyphenylhydrazine with 3-methyl-2-butanone in acetic acid (AcOH), affording the desired product in 60% yield. In the second step, quaternization of I1–2 using 1,4-butane sultone in toluene (PhMe) or acetonitrile (MeCN) afforded the sulfobutylated indolinium salts II1–2 in 65–70% yield. In the final step, a Knoevenagel condensation of II1–2 with substituted salicylaldehyde derivatives in ethanol (EtOH) delivered the target merocyanines 1–4 in moderate to good isolated yields after crystallization (50–59%). Compounds 1–4 were fully characterized by standard spectroscopic techniques, including mass spectrometry, ^1^H and ^13^C NMR spectroscopy, and X-ray crystallography (see the SI). X-ray-quality crystals of novel compounds 2–4 were grown by slow evaporation of the solvent over a period of 3–4 days, using EtOH for compounds 2 and 4, and a MeOH–hexane mixture for compound 3. Structural analysis confirmed that all the compounds 2–4 were isolated in their open protonated merocyanine configuration (Scheme 1).

**Scheme 1:**
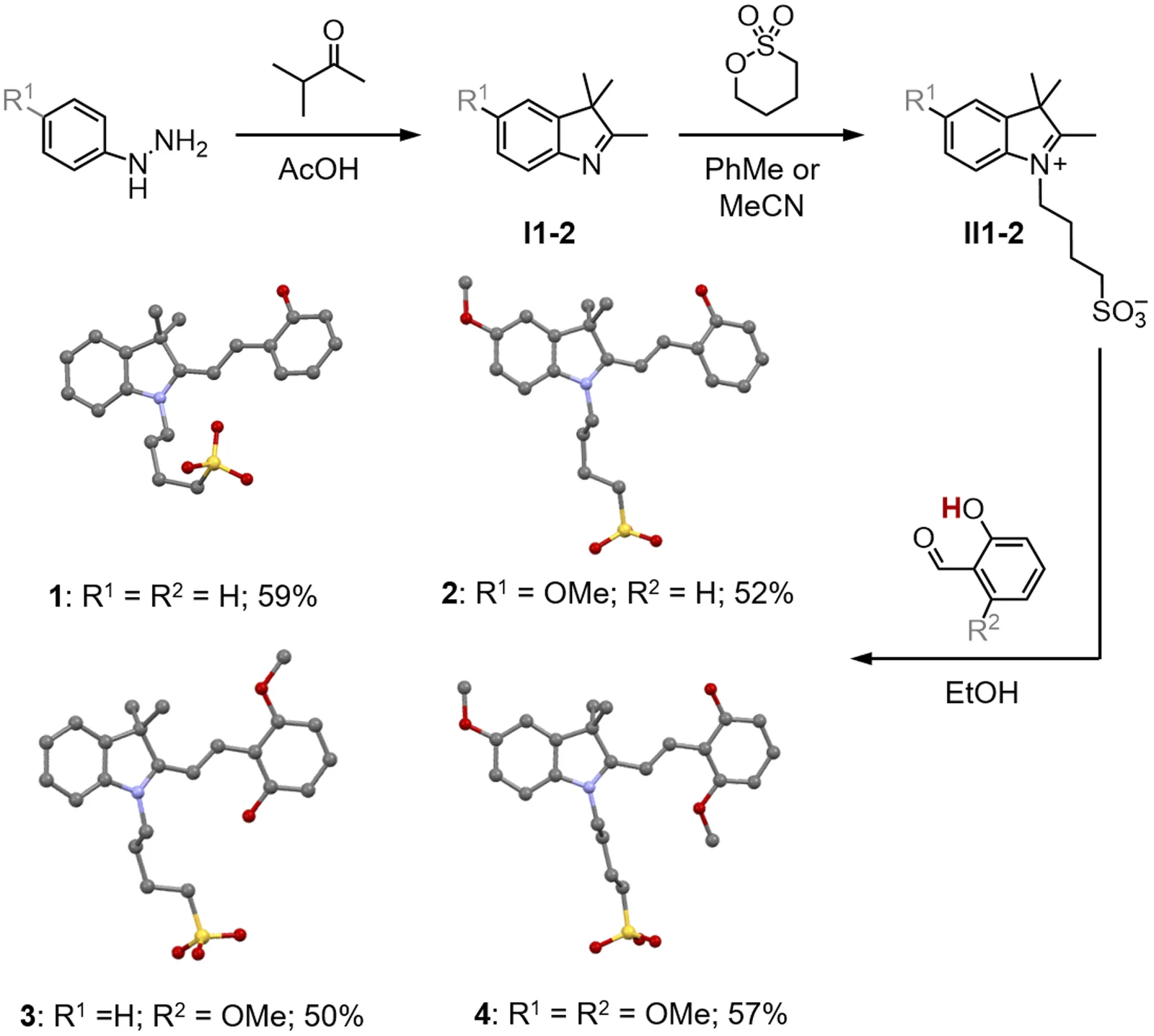
Synthesis of compounds 1–4. The structure of the products is based on single crystal X-ray analysis. Hydrogen atoms and solvent molecules are omitted for clarity. The X-ray structure of compound 1 is shown as an example based on the structure reported in ref. 25 for comparison.

As described by the four-state cyclic model (Fig. 1a), the ground-state equilibrium composition comprises of *trans*-MCH, MC and SP states – *i.e.*, *K*_a_^GS^ = *K*_a_(1 + *K*_c_). Thus, the ground-state apparent acid dissociation constants (p*K*_a_^GS^) were determined by monitoring the composition of 1–4 sample solutions in the dark at different pH values, after allowing sufficient equilibration to ensure that the recorded spectra correspond to thermodynamic equilibrium conditions. The optical readout after equilibration (*A*_eq_) was plotted as a function of the pH, and p*K*_a_^GS^ was determined by nonlinear least-squares curve fitting to the Boltzmann equation:1
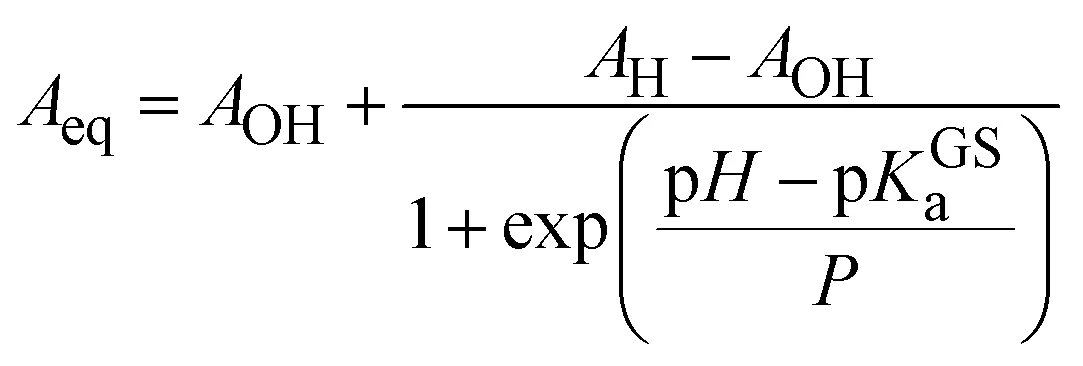
where *A*_H_ and *A*_OH_ are absorbances detected at low and high pH, respectively, whereas *P* represents a fitting factor.^43^ The corresponding UV-Vis spectra for 4 are shown in Fig. 2a; spectra obtained for compounds 1–3 are provided in Fig. S1 (see the SI). Comparison of the calculated p*K*_a_^GS^ values indicates that *para*-methoxy substitution on the indolinium moiety in compounds 2 and 4 leads to a lower acidity constant (higher p*K*_a_^GS^) relative to unsubstituted compound 1, whereas methoxy substitution on the chromene moiety results in negligible changes (Fig. 2b).

**Fig. 2:**
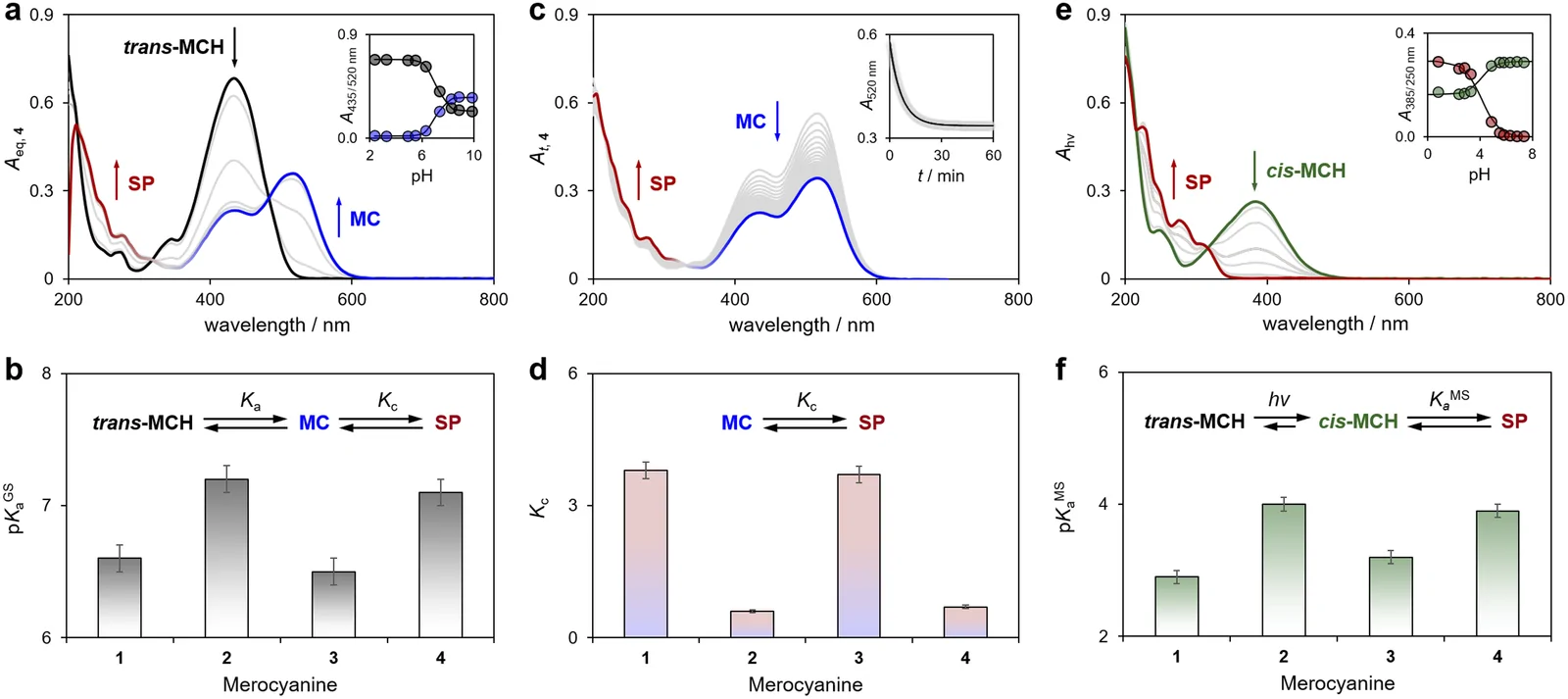
Thermodynamics of 1–4 UV-Vis absorption spectra of 4 at equilibrium, (a) recorded as a function of pH in the dark, (c) recorded at pH 10 as a function of time, and (e) under continuous 500 nm light irradiation with insets depicting the corresponding elaborations. Comparison of (b) the ground-state acidity constants, (d) the MC ⇌ SP isomerization constants, and (f) the metastable-state acidity constants for 1–4.

The MC ⇌ SP isomerization was then investigated by monitoring the decrease in absorbance at the *λ*_max_ of the MC species over time after mixing freshly prepared solutions of compounds 1–4 with a buffer at pH 10. The evolution of the UV-Vis spectrum for compound 4 is shown in Fig. 2c, while the corresponding kinetic traces for compounds 1–4 are provided in Fig. S2 (see the SI). The kinetic traces were fitted using a first-order decay model,^25^ which provides coefficients for estimating the equilibrium constant of the MC ⇌ SP interconversion (*K*_c_). The time dependent absorbance was described as:

2 *A*_t_ = *A*_eq_ + (*A*_0_ − *A*_eq_)e^(−*k*_obs_)*t*^

where *A*_t_, *A*_0_, and *A*_eq_ are the absorbances of MC at *λ*_max_ at any time *t*, at *t* = 0, and at equilibrium, respectively, while *k*_obs_ is the first-order rate constant of the first-order reaction. *K*_c_ was then determined as follows:3
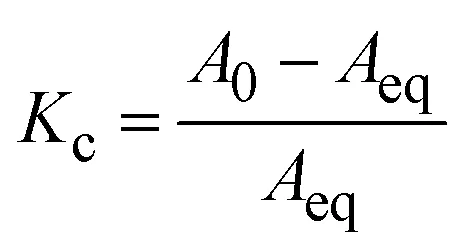


Merocyanines 1 and 3 show an average *K*_c_ of 3.7 ± 1.3 (Table S1), a value roughly sixfold greater than that obtained for compounds 2 and 4 (*K*_c_ = 0.6 ± 0.1). This trend suggests that *para*-substitution on the indolinium unit, as present in compounds 2 and 4, hinders the formation of the ring-closed SP isomer (Fig. 2d).

Consequently, the equilibrium is strongly biased toward the open, deprotonated merocyanine state MC. To gain molecular insights into the experimentally observed substituent dependence of the MC ⇌ SP equilibrium, density functional theory calculations were performed on both the open merocyanine (MC) and closed spiropyran (SP) forms of the four derivatives (see the Computational Methods and SI for details). In the gas phase, formation of the closed SP form is strongly favored while inclusion of aqueous solvation shifts the relative stability toward the more polar MC form, reducing the calculated reaction free energies to −2.5, −1.5, −2.1, and −0.8 kcal mol^−1^ for compounds 1–4, respectively (Table S2). Although the absolute calculated free energies remain sensitive to the implicit treatment of solvation, the results support the thermodynamic trend found experimentally. Ring closure is more favorable for compounds 1 and 3, with an average Δ*G*_MC→SP_ of −2.3 kcal mol^−1^, than for the indolinium-substituted compounds 2 and 4, for which the corresponding average is −1.1 kcal mol^−1^. Thus, introduction of a *p*-methoxy substituent on the indolinium unit increases the free energy of MC-to-SP conversion by approximately 1.1 kcal mol^−1^, which is remarkably consistent with the approximately sixfold variation in the experimental *K*_c_ values. This effect is predominantly electronic, because the thermal contribution opposes ring closure by a similar amount for all four derivatives, approximately 2.0–2.4 kcal mol^−1^, without altering their relative ordering. To rationalize this substituent effect, natural bond orbital (NBO) analysis was performed for the open merocyanine and closed spiropyran forms of the doubly substituted compound 4. NBO analysis represents the calculated electron density in terms of localized bonds, lone pairs, and donor–acceptor interactions. Substantial methoxy-to-arene π donation was identified at both substitution sites. Although the corresponding second-order perturbation stabilization energies increased by similar amounts upon ring opening, the R^1^ = OMe showed a more pronounced shortening of the aryl–O bond, increase in the Wiberg bond index, and depletion of its p-type oxygen lone pair instead of the *R*^2^ methoxy group. These changes are small but internally consistent (Table S3), providing qualitative evidence that R^1^ = OMe becomes more strongly conjugatively engaged in the open merocyanine, whereas the conjugative involvement of R^2^ = OMe changes only modestly between the two isomeric forms. This differential response is consistent with preferential stabilization of the open form by substitution at R^1^.

We then studied the acid–base properties of compounds 1–4 under steady light irradiation by means of UV-Vis spectroscopic titrations performed over a range of pH values (Fig. 2e, MCH 4 and Fig. S3, MCHs 1–3). Under steady light irradiation, the system exists as a photochemical equilibrium comprising the *cis*-MCH and SP isomers. Metastable-state apparent acid dissociation constants (p*K*_a_^MS^) of 1–4 were determined from the profiles of the absorbance of either *cis*-MCH or SP (Fig. S3) as a function of the pH by non-linear least–squares curve fitting to eqn (1), with the measurements performed under continuous irradiation at 500 nm to establish the metastable-state photostationary state. Analysis of the resulting trend showed that *para*-methoxy substitution on the indolinium moiety leads to a marked increase in the p*K*_a_^MS^ values, whereas substitution on the chromene moiety has only a minimal influence (Fig. 2f). This behavior mirrors the trend observed for the ground state acidity (Fig. 2b). As a result, the photoacidity parameter remains nearly unchanged (*Π* ≈ 3.2) across the series, indicating that these substitutions influence the ground and metastable states to a similar extent without significantly altering the overall photoacidic response.

After having assessed the thermodynamics of the system, we moved on to study the kinetics of compounds 1–4. First, we monitored the photoisomerization kinetics of MCH to SP under light irradiation by LED-coupled UV-Vis spectroscopy (see Fig. S6 for the experimental setup).^44^ Kinetic analysis was performed according to the following equation:4
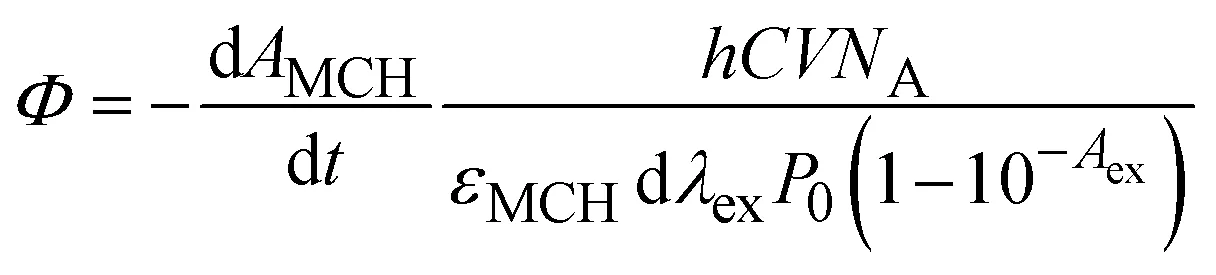
where *Φ* represents the quantum yield of the isomerization reaction, d*A*_MCH_/d*t* and *ε*_MCH_ are the rate of absorbance depletion and the molar absorptivity of the MCH form, and *h*, *C*, *V*, *N*_A_, *P*_0_ and *d* are the Planck constant, the concentration, the volume of the sample, the Avogadro number, the power of the LED light source and the optical path length of light through the sample, respectively. *λ*_ex_ is the excitation wavelength, while *A*_ex_ is the absorbance of the photochemical system at *λ*_ex_. To calculate *ε*_MCH_, the exact concentrations of the MCH 1–4 samples were first determined *via* potentiometric titrations prior to UV-Vis concentration-dependent analyses at pH 4 (Fig. S4). *ε*_MCH_ is the slope of the corresponding absorbance *vs.* concentration calibration curve (Fig. S5). The kinetic traces obtained for compounds 1–4 were analyzed by fitting the decay profiles using the slope (Fig. 3a, MCH 4 and Fig. S7, MCHs 1–3). Compounds 2 and 4 exhibit quantum yields that are comparable to the values previously reported for reference merocyanine systems (*e.g.*, Liao's merocyanine *Φ* = 0.3823). In contrast, compounds 1 and 3 show slightly lower *Φ* values of 0.23 and 0.13, respectively, indicating reduced efficiency of photoinduced proton release in these derivatives (Fig. 3b).

**Fig. 3:**
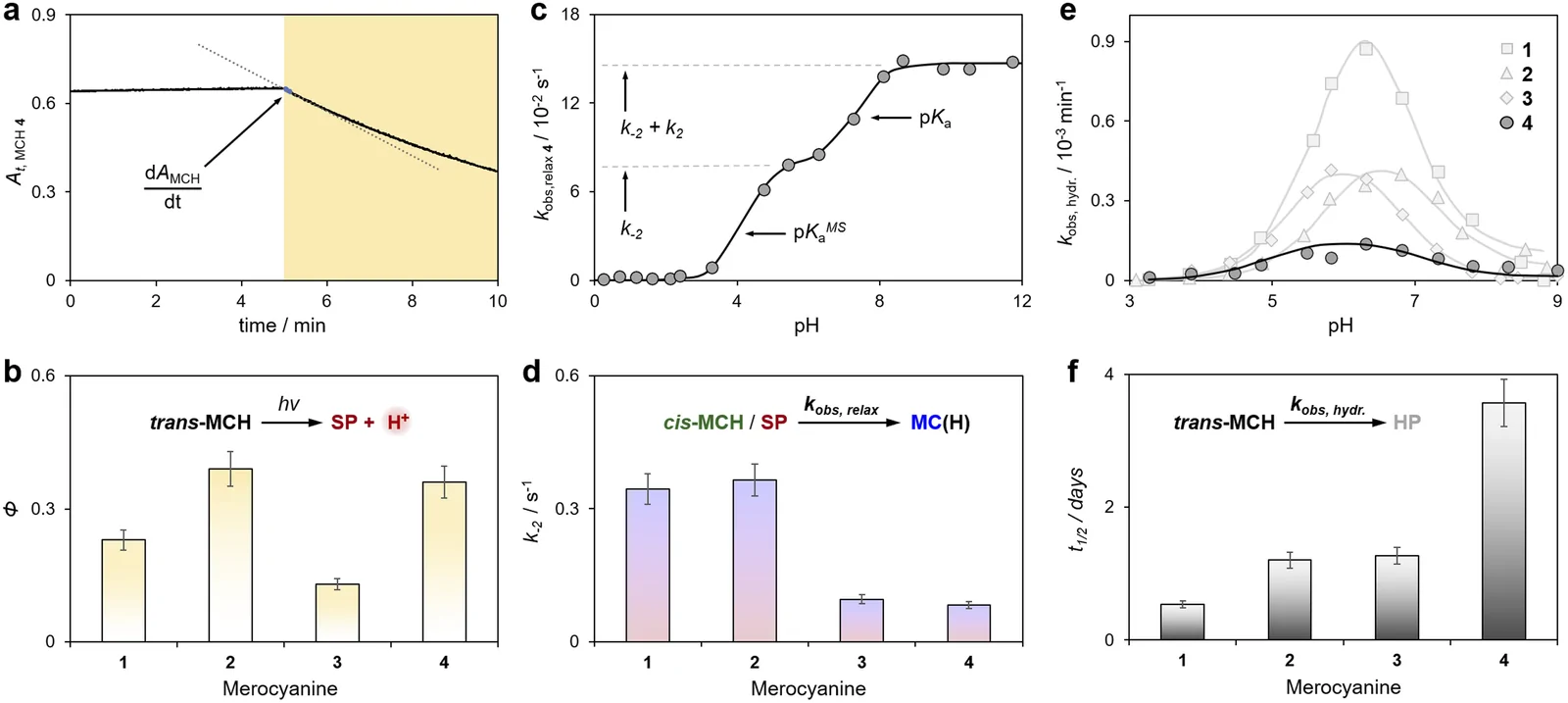
Kinetics of 1–4 (a) Absorbance depletion obtained for 4 under 425 nm LED light irradiation. The slope of the linear fit (d*A*_MCH_/d*t*, dotted line) is used in eqn (4) to calculate the quantum yield. (c) Relaxation profile of 4 as a function of pH after light irradiation; the solid black line represents the best fit to eqn (5). (e) Profiles of apparent first-order rate constant for the hydrolysis of compounds 1–4 as a function of pH; the solid lines represent the best fits to eqn (6). Comparison of (b) the quantum yields of isomerization, (d) the backward rate constants (*k*_−2_), and (f) the hydrolysis half-lives in water of 1–4

We monitored the relaxation kinetics of the system under dark conditions and found that the observed rate constant of relaxation (*k*_obs_,_relax_) gradually increased with increasing pH (Fig. 3c, MCH 4), following characteristic double-sigmoidal behaviors with two inflection points near the respective p*K*_a_ values. In all cases, the profiles obtained experimentally are in line with the following equation:^25^5
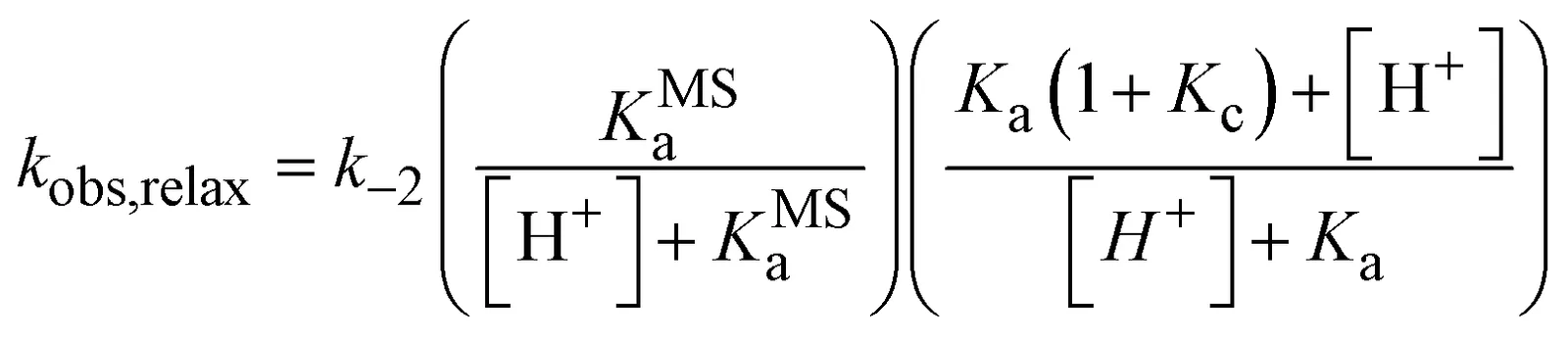
which reflects the ability of MCHs 1–4 to undergo reversible proton release and uptake through a four-state cyclic interconversion mechanism. The optimized parameters resulting from fitting are in line with the set of thermodynamic and kinetic constants determined above (see Fig. S8).

The set of kinetic parameters extracted from the analysis of the experiments indicate that ring closing and ring opening are governed by different substituent effects. The apparent ring-closing rate constant *k*_2_ is affected by substitution at both *R*^1^ and *R*^2^ sites (1.7, 0.2, 0.4, and 0.06 s^−1^ for compounds 1–4) whereas *k*_−2_ is only weakly affected by *R*^1^ but decreases by approximately four- to fivefold upon methoxy substitution at *R*^2^ (Fig. 3d). This distinct sensitivity suggests that the observed SP–MC interconversion does not correspond to a single elementary C–O bond-forming or bond-breaking event but instead involves additional conformational reorganization through one or more cisoid structures, possibly stabilized by specific interactions with the protic solvent. At the DFT-SMD implicit-solvent level, we located a structurally similar transition state (TS) for compounds 1–4 (Fig. S12). Displacement along its imaginary mode led toward the *trans*-merocyanine basin in one direction. In the opposite direction, the structures relaxed directly to SP for compounds 1 and 3, whereas relatively high-energy cisoid minima were obtained for compounds 2 and 4 (see the Computational methods for details and Table S4). Consistent with the effect of *R*^1^ on *k*_2_ found experimentally, the SMD calculations show that –OMe raises the free-energy barrier from the *trans*-merocyanine to the TS by 0.58–1.12 kcal mol^−1^ for fixed *R*^2^ (see Table S5), indicating that substitution at this position hinders the first activated step leading away from the *trans* form toward cyclization. The calculated *R*^2^ effect, however, does not reproduce the experimental trend on *k*_-2_ when the TS is assumed to connect directly to SP, showing that the TS cannot be regarded as a common transition state for the complete SP–MC interconversion. The identity and energetic role of the species formed immediately after C–O bond cleavage therefore remain uncertain. This limitation is likely particularly important for the opening process, in which specific hydrogen bonding and solvent reorganization in the protic medium may decisively stabilize cisoid, charge-separated configurations that are not adequately described by the implicit-solvent model.

A critical factor limiting the application of MCHs in aqueous environments is their intrinsic susceptibility to irreversible hydrolysis. This process involves nucleophilic addition of water to the ene-iminium bond followed by decomposition of the resulting tetrahedral intermediate to yield the corresponding indolinium and salicylaldehyde derivatives.^25^ Subsequently, the hydrolytic stability of 1–4 was examined within the pH range of 3 to 9 (Fig. S9). Consistent with a Schiff base-like mechanism,^45,46^ the hydrolysis kinetics display a characteristic pH-dependent behavior, resulting in bell-shaped profiles (Fig. 3e) for all compounds. The observed rate constant of hydrolysis can be determined by the following equation:6
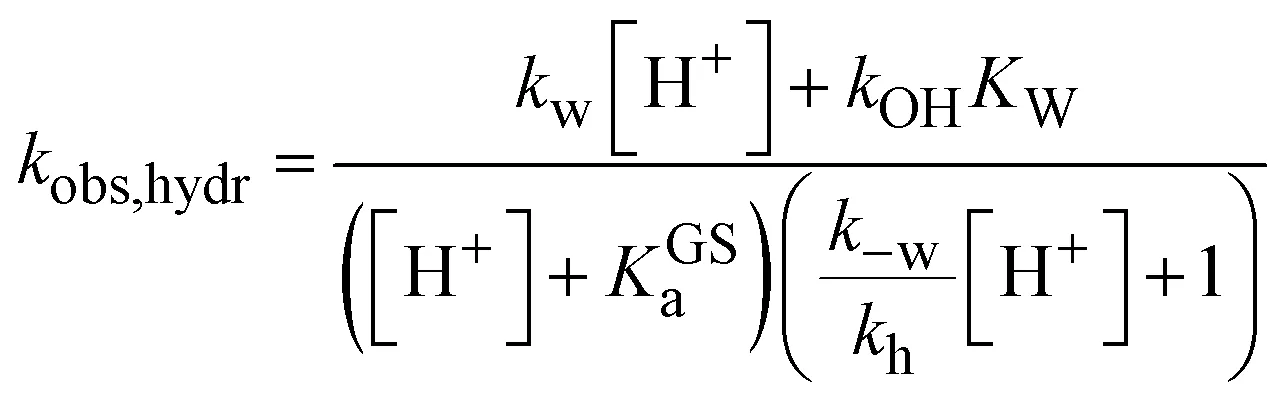
where *k*_w_ and *k*_−w_ represent the forward and backward rate constants for the nucleophilic attack of water, *k*_OH_ the rate constant for the attack of the hydroxide ion, and *K*_w_ is the autoprotolysis constant of water. At high pH values, hydroxide ions become the predominant nucleophile, but nucleophilic attack is strongly disfavored as the γ-carbon becomes less electrophilic following ring closure of MC to form SP.^25^ The obtained profiles clearly indicate that the doubly substituted derivative 4 (black curve) exhibits a markedly reduced tendency toward hydrolytic degradation in aqueous media, outperforming the other derivatives 1–3 (Fig. 3e). The corresponding half-life values are shown in Fig. 3f for comparison. The number of methoxy substituents directly conjugated to the ene-iminium core directly enhances hydrolytic stability. In particular, the *bis*-substituted derivative 4 exhibited the longest half-life (3.5 days), compared with the mono-substituted derivatives 2 and 3 (1.3 days) and the unsubstituted compound 1 (0.5 days). The thermodynamic and kinetic parameters obtained for compounds 1–4, highlighting the effect of methoxy substitution, are systematically summarized in Table S1.

Finally, we examined the ability of the best performing merocyanine 4 to sustain reversible light-triggered proton release. To harness the maximum achievable concentration of 4 in its MCH isomeric form, solubility measurements were carried out in a 40% MeOH/H_2_O (v/v) mixture. The resulting solubility (*S*_MCH_) of MCH 4 was found to be ∼1.02 mM by UV-Vis spectroscopy (Fig. S10). Having established the accessible concentration range, we investigated the performance of MCH 4 for light-controlled proton release (Fig. 4a) by carrying out pH-jump experiments (refer to Fig. S11 for the experimental setup) with a solution of 4 neutralized at pH = p*K*_a_^GS^. Upon light irradiation at 500 nm, the pH drops by three units and consequently, MCH 4 produces a reversible pH change of approximately 3.2 units (Fig. 4b), while retaining stable performance over 87 switching cycles run over two days.

**Fig. 4:**
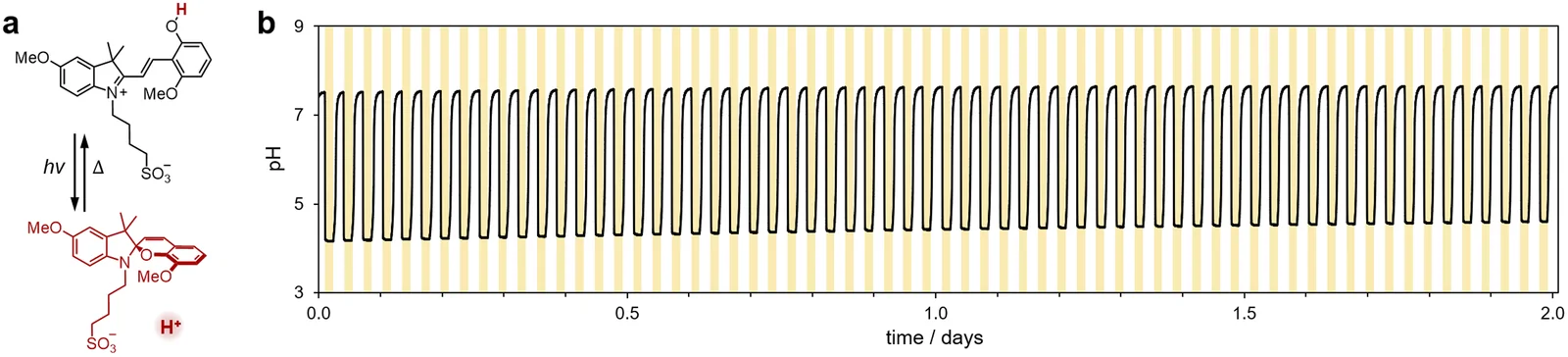
(a) Schematic representation of the reversible proton release by MCH 4 in water. (b) Reversible pH-jump dynamics obtained for a solution of 4 neutralized at pH = p*K*_a_^GS^. Yellow-shaded regions indicate the time intervals during which the light source was active. Experimental conditions: [4] = 1 mM, [KCl] = 20 mM in 40% MeOH/H_2_O, *T* = 25 °C; irradiation: 500 nm; cycles (light/dark) = 15 min/30 min. With this solvent composition and within the pH range tested, both *k*_obs_,_relax_ and *k*_obs,hydr_ change only marginally compared to pure water.^17^

## Conclusions

Collectively, our results demonstrate that mesomeric tuning through strategic methoxy substitution provides an effective alternative approach to optimize the performance of merocyanine photoacids in water. Increasing the number of methoxy groups directly conjugated to the ene-iminium core progressively enhances hydrolytic stability, with the bis-substituted derivative 4 exhibiting a nearly three-fold increase in half-life compared to the mono-substituted analogues, and a seven-fold improvement relative to 1. In addition, compound 4 maintains a pronounced photoacidic response, undergoing a light-induced decrease in acidity of approximately three pH units. The combination of enhanced aqueous stability and predictable photochemical switching enabled the development of a system capable of reversible visible light-controlled pH modulation with pH jumps of 3.2 units, sustained over multiple consecutive cycles for more than two days of operation. Our results further reveal a distinct structure–property relationship, in which *para*-methoxy substitution on the indolinium moiety predominantly modulates the thermodynamic parameters (p*K*_a_^GS^, p*K*_a_^MS^ and *K*_c_) whereas *ortho*-methoxy substitution on the chromene moiety primarily governs the kinetics of relaxation (*k*_obs,relax_). These findings establish mesomeric tuning as a promising strategy for developing robust photoacids for controlling aqueous-based dynamic chemical systems. We are currently integrating this new bis-substituted merocyanine skeleton into robust and scalable solar energy harvesting devices.

## Author contributions

C. P. conceived and supervised the project. B. Y. and S. E. C. carried out experiments and data elaboration. M. R. carried out X-ray diffraction and solved the crystal structures of 2–4. B. F. carried out computational studies. B. Y. wrote the manuscript with support from C. P. and B. F. All authors commented on it. ‡B. Y. and S. E. C. contributed equally to this work.

## Conflicts of interest

The authors declare no competing financial interest.

## Supplementary Material

SC-OLF-D6SC06885E-s001

## Data Availability

Supplementary information (SI): experimental methods, as well as NMR, X-ray, thermodynamic and kinetic data. See DOI: https://doi.org/10.1039/d6sc06885e. CCDC 2580635–2580637 contain the supplementary crystallographic data for this paper.^47*a*–*c*^
