# Supplementary material for: Mesomeric tuning of merocyanine photoacids
Source: Chem Sci. 2026 Oct 26. Online ahead of print. doi: 10.1039/d6sc06885e (PMC13701144; doi:10.1039/d6sc06885e)
Supplement: Supplementary file 1 [file SC-OLF-D6SC06885E-s001.pdf]

Supplementary Information (SI) for Chemical Science.
This journal is © The Royal Society of Chemistry

Supplementary Information

Mesomeric Tuning of Merocyanine Photoacids

Bharti Yadav[a‡*], Saif Eddine Cherif[a‡], Stefano Contena[b], Gianpiero Cera[c], Marzio Rancan[d], Barbara Fresch[e], Jack Li-Yang Chen[b,f,g], Cristian Pezzato[a*]

[a]Laboratory for Macromolecular and Organic Chemistry (MOC), Department of Chemical Sciences, University of Padova, via Marzolo 1, 35131 Padova, Italy

[b]Department of Biotechnology, Chemistry and Pharmaceutical Sciences, Università degli Studi di Siena, 53100 Siena, Italy

[c]Dipartimento di Scienze Chimiche, della Vita e della Sostenibilità Ambientale, Università di Parma, Parco Area delle Scienze 17/A, I-43124 Parma, Italy

[d]Institute of Condensed Matter Chemistry and Technologies for Energy (ICMATE), National Research Council (CNR), c/o Department of Chemistry, University of Padova, Via F. Marzolo 1, 35131 Padova, Italy

[e]Department of Chemical Sciences, University of Padova, via Marzolo 1, 35131 Padova, Italy

[f]School of Science, Auckland University of Technology, 34 St Paul St, Auckland, New Zealand

[g]MacDiarmid Institute for Advanced Materials and Nanotechnology, Wellington, New Zealand

[‡]authors contributed equally

Email: cristian.pezzato@unipd.it

1. General Information......3
   1.1 Experimental Methods......3
   1.2 Computational Methods......3
2. Synthetic Procedure......6
   2.1 General procedure for the synthesis of ***1-4***......6
3. Thermodynamic and kinetic parameters of **1-4**......9
4. Single crystal XRD experimental data of **2-4**......10

5. Acid dissociation constants of MCHs **1-4** (in dark conditions) 12

6. MC-to-SP Equilibration in the Dark of **1-4**... 13

7. Acid dissociation constants of MCHs **1-4** (in light conditions) 14

8. Potentiometric Titrations of **1-4**.. 15

9. Molar Extinction coefficients of **1-4**... 16

10. Isomerization under Light Irradiation (Quantum Yields of **1-4**)...................................... 17

11. SP-to-MC(H) relaxation in the dark of **1-4**... 20

12. Hydrolysis profiles of **1-4**... 21

13. Solubility and pH Jump Setup... 22

14. Computational Data... 24

15. ^{1}H, ^{13}C NMR and HRMS spectra of MCHs **1-4**.. 30

16. References.. 42

1. General Information

1.1 Experimental Methods

All reagents and solvents were purchased from commercial suppliers (Sigma-Aldrich, TCI, Acros Organics, Fluorochem, Abcr) and were used without further purification. Compounds **I2** and **II1-2** were synthesized and purified as described previously.[1-3] H_2O used in all preparations was filtered with a MilliQ-Integral5 purification system (mQ H_2O). Thin layer chromatography (TLC) was performed on silica gel plastic sheets bought from Sigma-Aldrich. All the NMR spectra acquired were analyzed with MNova software. Chemical shifts (δ) are reported in parts per million (ppm), while coupling constants (*J*) are given in Hertz (Hz). All the compounds were analyzed using HR-MS FFI performed on the Xevo Q-TOF (Waters, USA) mass spectrometer. The pH measurements were conducted using a Metrohm Titrando 888 coupled with a Biotrode glass electrode and processed using the software Metrohm Tiamo Light. Sample photoirradiation was carried out using a Prizmatix FC-LED-500Z high-power LED light source (425 or 500 nm). The light beam was delivered by polymer optical fibers (core 1500 μm, NA 0.63) either i) positioned orthogonally to and just below the liquid/air interface of sample solutions (UV-Vis, pK_a^{MS}), or ii) connected to an FCM1-06 collimator coupled with a 45° mirror cage, resulting in a light beam tilted by 90° (for pH jump experiments). For quantum yield measurements, see the description provided below (Figure S6). Power measurements of the fiber-coupled LED output were made with a Thorlabs S142C integrating sphere photodiode power sensor; the uncertainty is 5%. UV-

Vis spectra and kinetics were acquired on an Agilent Cary 60 spectrometer equipped with an 18-cell holder coupled to a Huber thermostat, using Suprasil quartz cuvettes (114-QS) from Hellma Analytics. Potassium phosphate buffers from pH 2 to 12 were prepared by titrating a solution of H_3PO_4 (100 mM, 1L) with 10 M KOH. Standard 0.1 N HCl was used for preparing samples below pH 2. All buffer stocks were stored at room temperature. To account for the pH change in sample preparations, the pH of each buffer stock was measured again after dilution, directly into cuvette samples. Each curve fitting was performed in Excel, using the Solver add-in to minimize the sum of the squared deviations between the experimental data and the corresponding model function.

1.2 Computational Methods.

Density functional theory calculations were performed using Gaussian 16, Revision B.01.[4] The four substitution patterns were represented as neutral closed-shell singlets. The computational models retained the photochromic merocyanine/spiropyran framework and the corresponding methoxy substitution pattern, whereas the terminal sulfonate group present in the experimentally investigated compounds was not included. Geometry optimizations and harmonic vibrational-frequency calculations were carried out at the ωB97X-D/6-31+G(d,p) level of theory, both in the gas phase and in aqueous solution. Solvent effects were described using the SMD implicit-solvation model[5] for water, with full reoptimization of the molecular geometries in the solvent environment. Harmonic frequency calculations were performed at the same level of theory as the corresponding geometry optimizations. Local minima were identified by the absence of imaginary frequencies, whereas transition structures were characterized by a single imaginary frequency associated with the relevant reaction coordinate. The reported Gibbs free energies were calculated as the sum of the electronic energy and the corresponding thermal correction. For each derivative, one representative transoid merocyanine (MC) conformer and one closed spiropyran (SP) conformer were considered. Although the open merocyanines exhibit considerable conformational flexibility, only a single representative conformer was considered for each compound. Importantly, the same conformational arrangement was maintained throughout the

series, with only the substituents being varied. Consequently, the calculations are not intended to reproduce the absolute MC/SP equilibrium free energies, which are expected to depend on conformational sampling and solvent stabilization of the highly polar merocyanine form, but rather to isolate the intrinsic electronic contribution of the substituents. allowing a direct comparison of the electronic effect of the different methoxy substitution patterns on the relative stability of the two isomers. Natural bond orbital (NBO) analyses[6-7] were performed on the optimized open merocyanine and closed spiropyran structures using the same B97X-D/6-31+G(d,p)/SMD (water) level of theory employed for geometry optimizations. Natural population analysis (NPA) charges, Wiberg bond indices, and second-order perturbation energies associated with donor–acceptor interactions were evaluated to assess the electronic effects of methoxy substitution, and particularly the extent of methoxy-to-arene conjugative donation in the two isomeric forms. The thermal MC-to-SP reaction pathway was investigated by first examining the conformational conversion of the transoid MC structure into a cisoid geometry suitable for ring closure in the gas phase. Relaxed potential-energy scans were performed along the central C–C–C–C torsional coordinate of the polymethine chain. The selected dihedral angle varied in 12 increments of 15° while all remaining geometrical parameters were optimized at each point (Figure S13). The structures of the 8th step belonging to maximum-energy region of each relaxed scan were used as initial guesses for transition-state optimizations. The resulted gas phase transition state structures were subsequently reoptimized in SMD water to obtain the relevant activation free energy. To investigate the reaction path, the nature of the transition structure was examined by generating displacements from the transition state in both directions along the intrinsic reaction coordinate, and the structures obtained on the two sides of the saddle point were subsequently optimized without geometrical constraints and subjected to frequency analysis. These optimizations converged to the transoid MC minimum on one side and to the closed SP minimum on the other side in the case of compounds **1**-**2**, while the (high energy) stable cisoid MC form was found for compounds **3**-**4**.

2. Synthetic Procedures

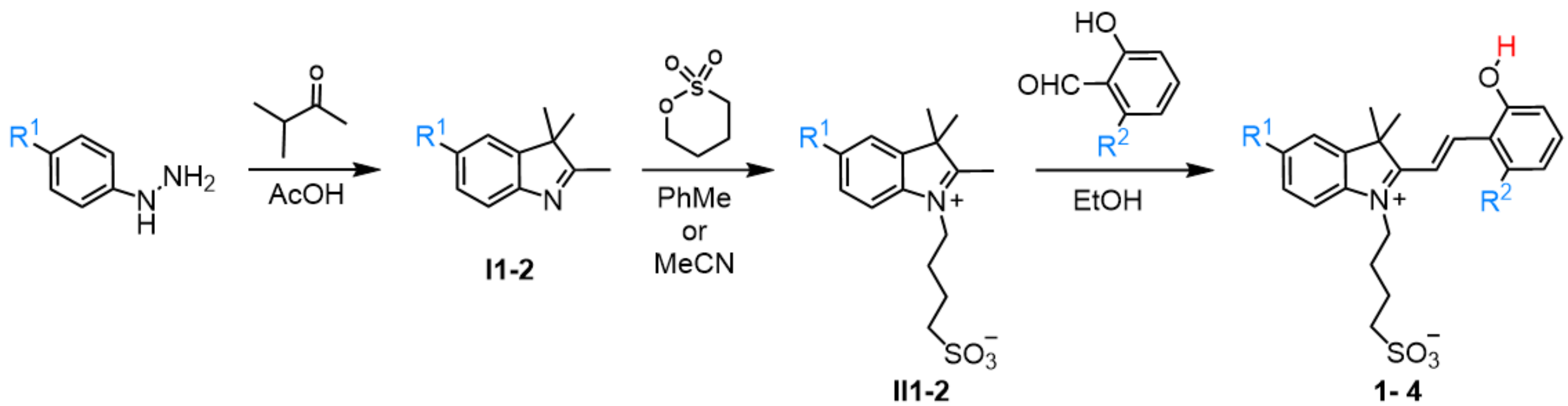

Entry	R^1	R^2
**1**	H	H
**2**	OMe	H
**3**	H	OMe
**4**	OMe	OMe

Scheme S1. Synthetic scheme for the library of compounds **1-4**.

*2.1 General procedure for the synthesis of **1-4**.*

In a 50-mL round-bottom flask, 2,3,3-trimethylindolium-propyl-sulfonates (**II1-2**) were dissolved in absolute EtOH (ca. 20 mL/g). The corresponding salicylaldehyde (1.3 equivalents) was added, the flask sealed with a septum and the solution degassed by gentle N_2 bubbling (10 min) prior to heating up to 90 °C under stirring for 24 h. The resulting orange precipitate was filtered, washed thoroughly with EtOH to remove unreacted materials and recrystallized from hot MeOH. Crystals were collected, washed with cold MeOH and dried under vacuum (isolated yield ≥ 50%). X-ray-quality crystals were obtained by using EtOH and MeOH/hexane mixture.

1. The general procedure depicted above was followed.[3] Indolinium **II1** (1.00 g, 3.43 mmol, 1.00 eq.) was dissolved in anhydrous ethanol (0.17 M, 20.0 mL) and salicylaldehyde (475 μL, 544 mg, 4.45 mmol, 1.30 eq.) was added. The crude solid was recrystallized from hot methanol. The crystals obtained were collected by filtration, washed with cold methanol, and dried under vacuum to afford the product as an orange solid (59% yield).

^{1}H NMR (400 MHz, DMSO-d_6) δ 11.14 (s, 1H), 8.56 (d, *J* = 16.3 Hz, 1H), 8.20 (dd, *J* = 7.9, 1.6 Hz, 1H), 8.02 - 7.98 (m, 1H), 7.86 (s, 1H), 7.61 (dt, *J* = 5.5, 2.1 Hz, 2H), 7.49 (ddd, *J* = 8.6, 7.1, 1.6 Hz, 2H),

7.07 - 7.02 (m, 1H), 7.00 (t, *J* = 7.6 Hz, 1H), 4.63 (t, *J* = 7.7 Hz, 2H), 2.53 (d, *J* = 7.2 Hz, 2H), 1.98 (t, *J* = 6.7 Hz, 2H), 1.82 (d, *J* = 7.3 Hz, 2H), 1.77 (s, 6H). **^{13}C NMR** (101 MHz, DMSO- d_6) δ 181.7, 159.1, 149.2, 143.4, 140.8, 135.6, 130.4, 129.1, 123.0, 121.3, 116.7, 115.3, 111.6, 51.9, 50.2, 46.5, 27.0, 26.4, 22.3. **HRMS** (ESI/QTOF) m/z: $[M+H]^+$ Calcd for $C_{22}H_{26}NO_4S^+$ 400.1577; Found 400.1638.

2. The general procedure depicted above was followed.[8] Indolinium **II2** (1.00 g, 3.08 mmol, 1.00 eq.) was dissolved in anhydrous ethanol (0.15 M, 20.0 mL) and salicylaldehyde (426 μL, 488 mg, 4.00 mmol, 1.30 eq.) was added. The reaction mixture was stirred under the same conditions as described above. The crude solid was recrystallized from hot ethanol. The crystals obtained were collected by filtration, washed with cold ethanol, and dried under vacuum to afford the product as an orange solid (52% yield).

^{1}H NMR (400 MHz, DMSO-d_6) δ 11.04 (s, 1H), 8.46 (d, *J* = 16.4 Hz, 1H), 8.16 (d, *J* = 7.9 Hz, 1H), 7.91 (d, *J* = 8.9 Hz, 1H), 7.74 (d, *J* = 16.4 Hz, 1H), 7.51 (d, *J* = 2.4 Hz, 1H), 7.48 – 7.39 (m, 1H), 7.15 (dd, *J* = 8.9, 2.4 Hz, 1H), 7.03 (d, *J* = 8.3 Hz, 1H), 6.98 (t, *J* = 7.6 Hz, 1H), 4.60 (t, *J* = 7.7 Hz, 2H), 3.89 (s, 3H), 2.54 (d, *J* = 7.3 Hz, 2H), 1.97 (t, *J* = 7.8 Hz, 2H), 1.80 (d, *J* = 7.6 Hz, 2H), 1.76 (s, 6H). **^{13}C NMR** (101 MHz, DMSO-d_6) δ 180.4, 161.6, 159.7, 148.1, 146.6, 136.0, 135.0, 131.1, 122.3, 120.8, 117.5, 117.3, 115.8, 112.7, 109.7, 57.0, 52.7, 51.2, 47.4, 28.4, 23.2. **HRMS** (ESI/QTOF) m/z: $[M + H]^+$ Calcd for $C_{23}H_{28}NO_5S$+ 430.1683; Found 430.1761.

3. The general procedure depicted above was followed. Indolinium **II1** (1.00 g, 3.43 mmol, 1.00 eq.) was dissolved in anhydrous ethanol (0.17 M, 20.0 mL), and 6-methoxysalicylaldehyde (678 μL, 4.45 mmol, 1.30 eq.) was added. The reaction mixture was stirred under the same conditions as described above. The crude solid was recrystallized from methanol and hexane. The resulting crystals were collected by filtration, washed with cold methanol, and dried under vacuum to afford the product as an orange solid (50% yield).

^{1}H NMR (400 MHz, DMSO-d_6) δ 11.49 (s, 1H), 8.62 (d, *J* = 16.2 Hz, 1H), 8.11 - 7.81 (m, 3H), 7.60 (t, *J* = 6.9 Hz, 2H), 7.43 (t, *J* = 8.3 Hz, 1H), 6.66 (dd, *J* = 10.2, 8.4 Hz, 2H), 4.47 (s, 2H), 3.99 (s, 3H), 2.54 (t, *J* = 7.3 Hz, 2H), 1.99 (t, *J* = 7.8 Hz, 2H), 1.80 (d, *J* = 7.4 Hz, 2H), 1.75 (s, 6H). **^{13}C NMR** (101 MHz, DMSO-d_6) δ 182.6, 161.7, 161.2, 146.2, 143.5, 141.4, 137.0, 129.6, 129.3, 123.4, 115.4, 113.4, 111.4, 109.3, 103.0, 57.0, 52.0, 51.0, 47.0, 27.3, 27.2, 23.1. **HRMS** (ESI/QTOF) m/z: $[M+H]^+$ Calcd for $C_{23}H_{28}NO_5S^+$ 430.1683; Found 430.1761.

4. The general procedure depicted above was followed. Indolinium **II2** 1.00 g, 3.08 mmol, 1.00 eq.) was dissolved in anhydrous ethanol (0.17 M, 20.0 mL), and 6-methoxysalicylaldehyde (608 μL, 4.00 mmol, 1.30 eq.) was added. The reaction mixture was stirred under the same conditions as described above. The crude solid was recrystallized from hot ethanol. The resulting crystals were collected by filtration, washed with cold ethanol, and dried under vacuum to afford the product as an orange solid (57% yield).

^{1}H NMR (400 MHz, DMSO-d_6) δ 11.35 (s, 1H), 8.50 (d, *J* = 16.4 Hz, 1H), 7.94 (dd, *J* = 18.0, 12.7 Hz, 1H), 7.50 (d, *J* = 2.2 Hz, 2H), 7.38 (t, *J* = 8.3 Hz, 1H), 7.15 (dt, *J* = 15.3, 7.6 Hz, 1H), 6.65 (t, *J* = 7.5 Hz, 2H), 4.42 (t, *J* = 7.4 Hz, 2H), 3.98 (s, 3H), 3.87 (s, 3H), 2.54 (m, 2H), 1.98 (m, 2H), 1.79 (dd, *J* = 13.5, 6.2 Hz, 2H), 1.75 (s, 6H). **^{13}C NMR** (101 MHz, DMSO-d_6) δ 180.0, 161.0, 160.5, 160.4, 145.3, 143.8, 135.9, 134.3, 116.1, 114.7, 113.1, 110.9, 108.9, 102.5, 56.5, 56.1, 51.5, 50.5, 46.6, 27.0, 26.7, 22.6. **HRMS** (ESI/QTOF) m/z: $[M+H]^+$ Calcd for $C_{24}H_{30}NO_6S^+$ 460.1788; Found 460.1852.

See Figures S14-S25 for the full ^{1}H NMR, ^{13}C NMR and HRMS spectra.

3. Thermodynamic and kinetic data of 1-4.

Table S1: Summary of thermodynamic and kinetic parameters of 1-4.

Entry	R_1	R_2	K_c	pK_a^{GS}	pK_a^{MS}	Π	k_{-2} (min^{-1})	QY	ε_{MCH} ($L\ mol^{-1}\ cm^{-1}$)
**1**	H	H	3.8 ± 0.6	6.6 ± 0.1	2.9 ± 0.1	3.7 ± 0.2	0.345	0.23	32590
**2**	OMe	H	0.6 ± 0.1	7.2 ± 0.1	4.0 ± 0.1	3.2 ± 0.2	0.365	0.39	29062
**3**	H	OMe	3.7 ± 0.2	6.5 ± 0.1	3.2 ± 0.1	3.3 ± 0.2	0.096	0.13	41686
**4**	OMe	OMe	0.7 ± 0.1	7.1 ± 0.1	4.1 ± 0.1	3.2 ± 0.2	0.083	0.36	34759

4. Single crystal XRD experimental data for Compounds 2-4.

Data for compounds **2**-**4** were collected at low temperature (140-170 K) using a Bruker D8 Venture diffractometer equipped with Incoatec IμS3.0 (EF) microfocus sealed-tube (Cu-Kα, λ = 1.54178 Å), a Montel layer optics monochromator, and a Photon III C14 CPAD area detector. Data integration was done with SAINT, data scaling and absorption correction were performed with SADABS, in the APEX5 software.

The structures were solved with Olex2[9] by using ShelXT[10] structure solution program by Intrinsic Phasing and refined with the ShelXL[11] refinement package using least-squares minimization. In the last cycles of refinement, non-hydrogen atoms were refined anisotropically. Hydrogen atoms were included in calculated positions, and a riding model was used for their refinement. For structure **2**, the final Flack parameter was 0.35; consequently, the structure was refined as an inversion twin. For structure **4**, the final difference Fourier map revealed significant residual electron density peaks. The contribution of this disordered electron density was removed using the solvent-masking routine in OLEX2 (SQUEEZE algorithm). The calculation indicated a total solvent-accessible void volume of 660.4 $Å^3$ per unit cell (23.5% of the cell volume), containing 152 electrons per unit cell. This corresponds to approximately six ethanol molecules per unit cell (expected: 156 electrons).

Cambridge Crystallographic Data Centre (CCDC) numbers 2580635-2580637 contain the supplementary crystallographic data for this paper. These data are provided free of charge by the joint CCDC and Fachinformationszentrum Karlsruhe Access Structures service www.ccdc.cam.ac.uk/structures.

Table S2. Crystal data and structure refinement for compounds **2**-**4**.

Structure[a]	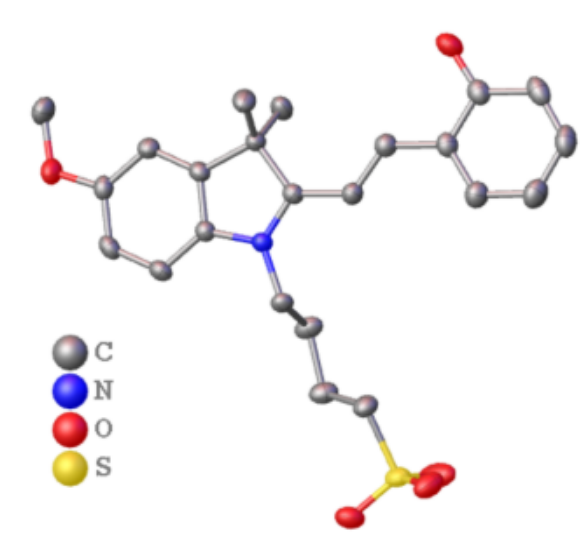	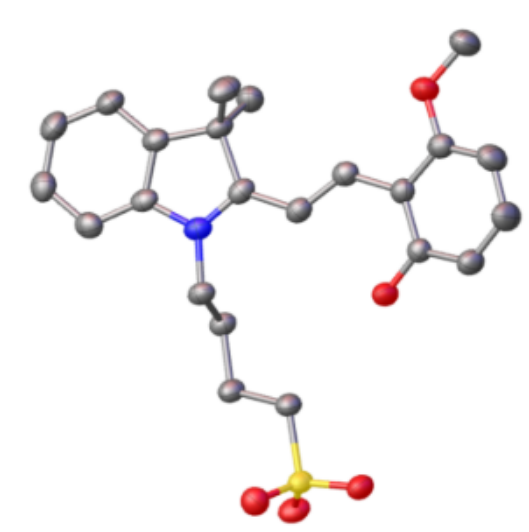	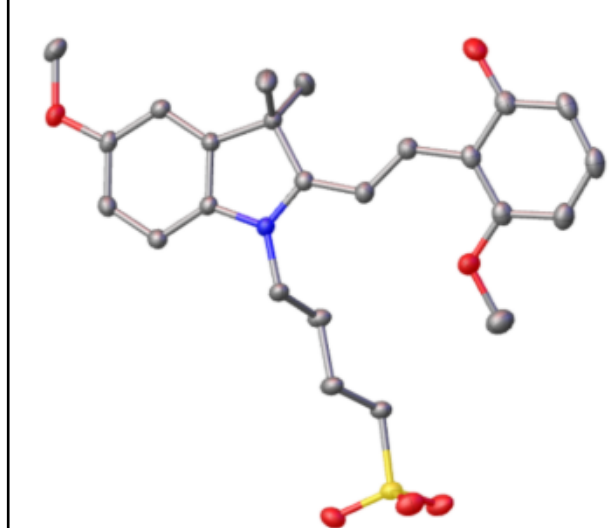
**Compound**	**2**	**3**	**4**
**Empirical formula**	$C_{25}H_{35}NO_7S$	$C_{23}H_{29}NO_7S$	$C_{24}H_{29}NO_7S$
**Formula weight**	493.60 (**2**·(EtOH + H_2O))	447.53 (**3**·H_2O)	477.56 (**4**·H_2O)
**Temperature (K)**	170	140	150
**Crystal system**	Orthorhombic	Orthorhombic	Monoclinic
**Space group**	$Pna2_1$	$P2_12_12_1$	$P2_{1/n}$
**a (Å)**	24.5738(17)	6.3950(5)	15.2363(8)
**b (Å)**	14.0415(8)	14.8219(12)	7.1485(4)
**c (Å)**	7.2648(4)	23.6495(18)	26.5545(13)
**α (°)**	90	90	90

β (°)	90	90	104.071(2)
γ (°)	90	90	90
Volume (Å^3)	2506.7(3)	2241.6(3)	2805.4(3)
Z	4	4	4
ρcalc (g/cm^3)	1.308	1.326	1.131
μ (mm⁻^{1})	1.523	1.616	1.347
F(000)	1056.0	952	1016.0
Crystal size (mm^3)	0.41 × 0.39 × 0.32	0.6 × 0.1 × 0.04	0.2 × 0.2 × 0.2
Radiation	Cu Kα (λ= 1.54178 Å)	Cu Kα (λ= 1.54178 Å)	Cu Kα (λ= 1.54178 Å)
2θ range for data collection/°	7.194 to 149.084	7.038 to 150.916	6.128 to 150.124
Index ranges (h,k,l)	-29 ≤ h ≤ 30, -17 ≤ k ≤ 17, -9 ≤ l ≤ 9	-8 ≤ h ≤ 7, -17 ≤ k ≤ 18, -29 ≤ l ≤ 29	-18 ≤ h ≤ 19, -8 ≤ k ≤ 8, -33 ≤ l ≤ 33
Reflections collected	5083	31051	5691
Independent reflections	5083 [R_{int} = 0.0432, R_{sigma} = 0.0260	4562 [R_{int} = 0.0786, R_{sigma} = 0.0397]	5691 [R_{int} = 0.0455, R_{sigma} = 0.0325]
Data / restraints / parameters	5083/2/316	4562 / 48 / 287	5691/247/306
Goodness-of-fit on F^2	1.078	1.055	1.028
Final R indexes [I ≥ 2σ(I)]	R_1 = 0.0362, wR_2 = 0.1032	R_1 = 0.0457, wR_2 = 0.1222	R_1 = 0.0421, wR_2 = 0.1150
Final R indexes [all data]	R_1 = 0.0364, wR_2 = 0.1033	R_1 = 0.0483, wR_2 = 0.1255	R_1 = 0.0438, wR_2 = 0.1154
Largest diff. peak/hole (eÅ⁻^{3})	0.58/-0.47	0.48 / -0.28	0.53/-0.45
CCDC number	2580636	2580635	2580637

[a]crystal structures are illustrated with thermal ellipsoids at 50% probability; solvent molecules and hydrogens are omitted for clarity.

5. Acid dissociation constants of MCHs 1-4 (in dark conditions)

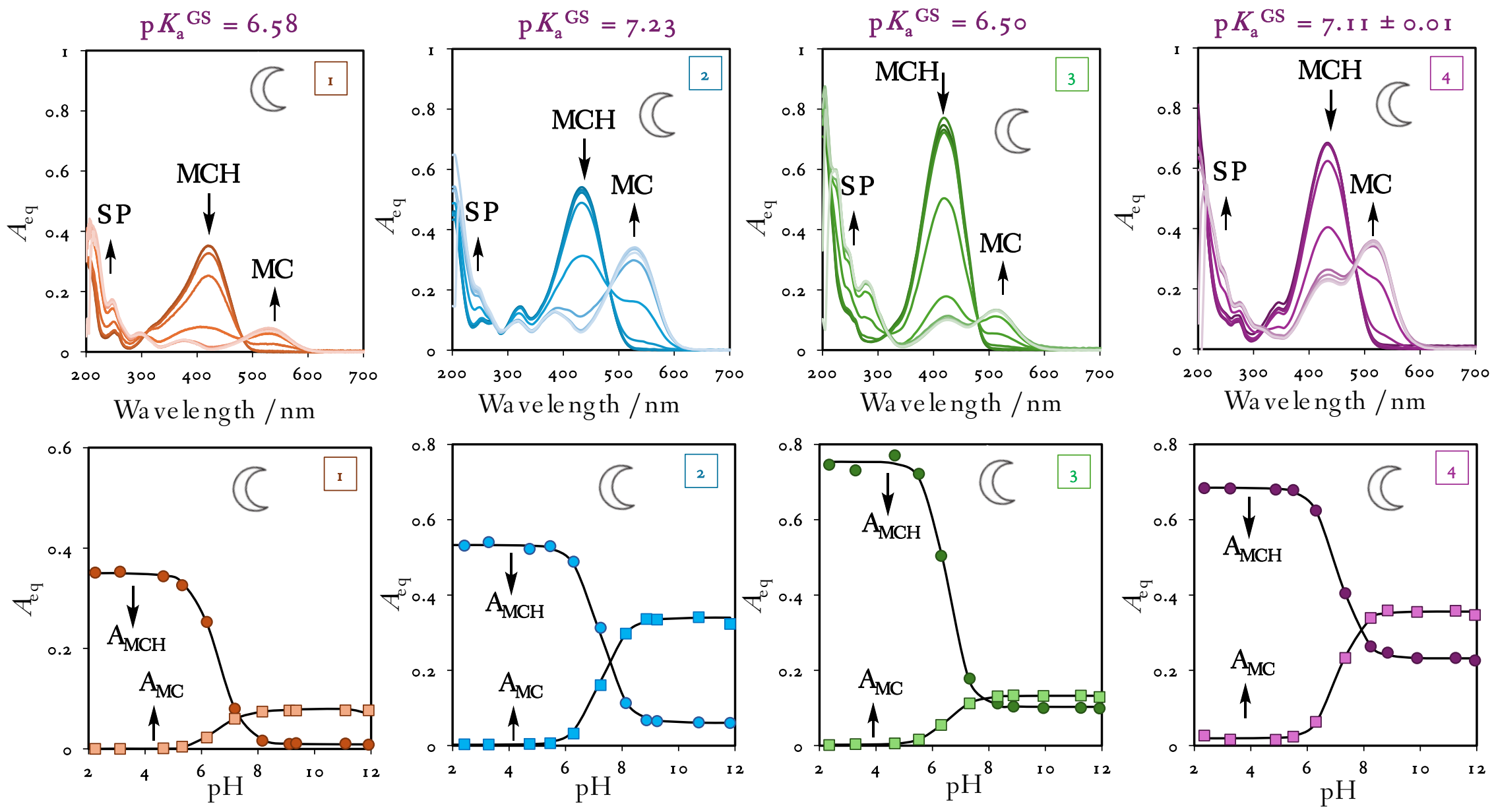

Figure S1. UV-Vis absorption titrations of MCHs **1**-**4** recorded in the dark over the pH range 2-12 (top) and the corresponding pH-dependent absorbance profiles of the MCH and MC species (bottom). Experimental conditions: [**1**-**4**] = 25 ± 2 μM, [phosphate buffers] = 20 mM, pH = 2-12, T = 25 °C.

6. MC to SP Equilibrium in the dark for compounds 1-4

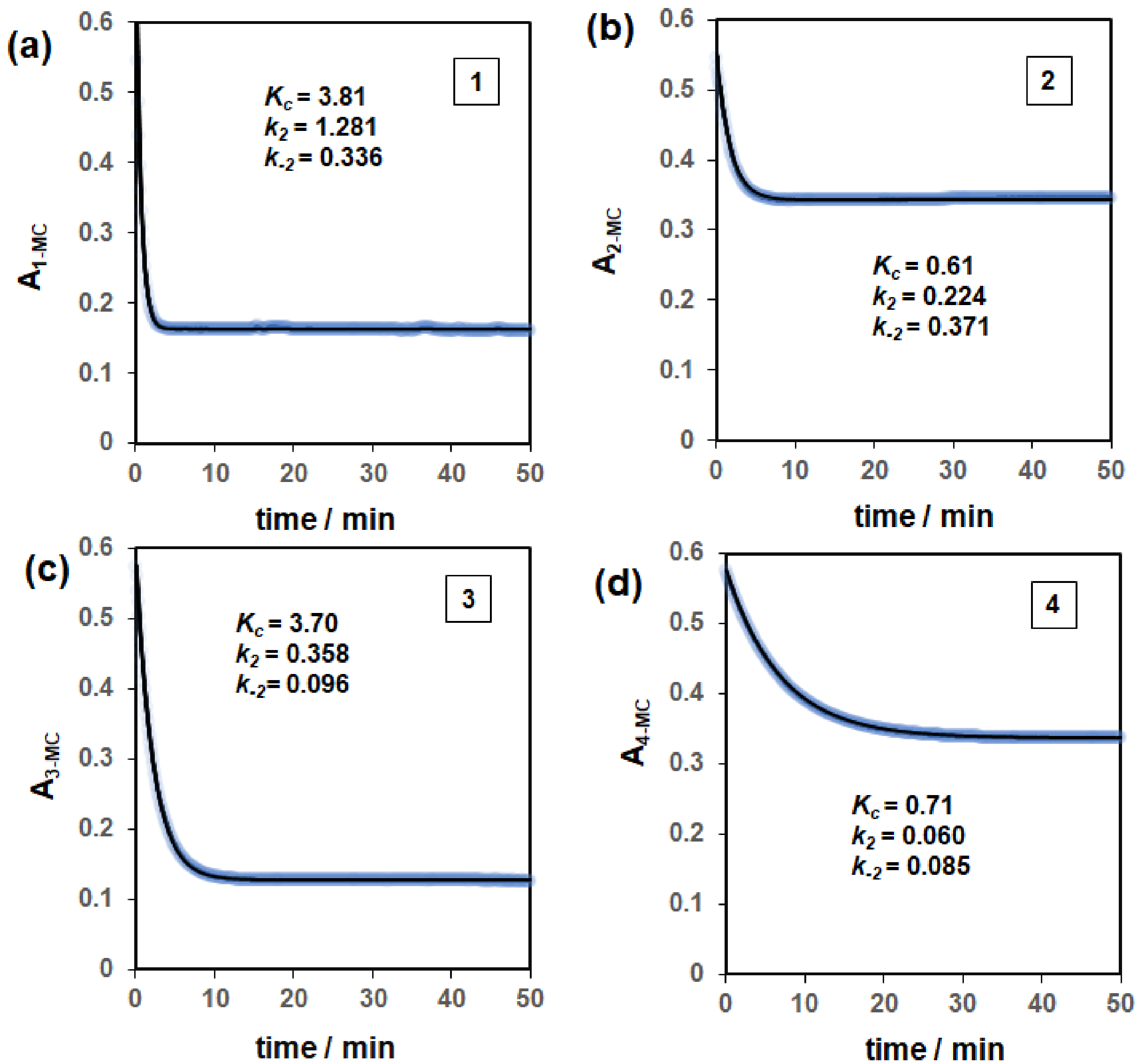

Figure S2. Kinetic profiles after on-site addition and rapid mixing of an aliquot (50 μL) of a solution (0.5 mM in mQ water) of compound **1** (top left), **2** (top right), **3** (bottom left) and **4** (bottom right) into a cuvette containing the buffer solution (950 μL); the solid black line represents the best fit to a first-order model equation 2. Experimental conditions: [**1**-**4**] = 25 ± 2 μM, [phosphate buffer] = 20 mM, pH = 10, T = 25 °C.

7. Acid dissociation constants of MCHs 1-4 (in light conditions)

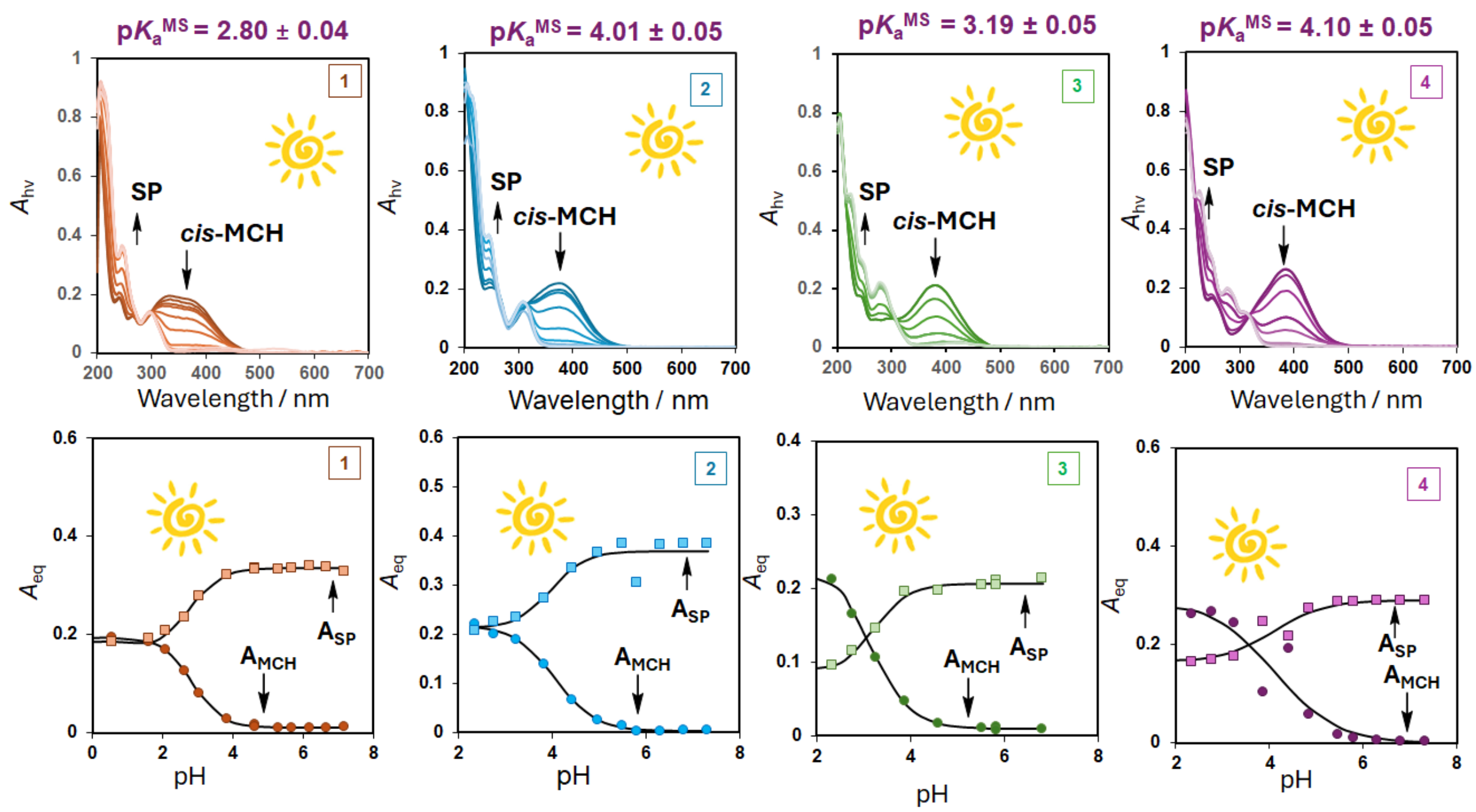

Figure S3. UV-Vis absorption titrations (top) of MCHs **1**-**4** monitored over time under continuous green light irradiation (λ= 500 nm) across a pH range and the corresponding metastable spectra (bottom).

Experimental conditions: [**1**-**4**] = 25 ± 2 μM, [phosphate buffers] = 20 mM (pH > 2), *T* = 25 °C.

8. Potentiometric Titrations of 1-4

The resulting pH vs. V_{NaOH} profiles were processed by calculating the second derivative curve, from which the equivalence point (V_{eq}) was extrapolated as the V_{NaOH} at $d^2pH/d\,V_{NaOH}^2 = 0$; this value matches the equivalence point automatically generated by the Metrohm Tiamo™ software.

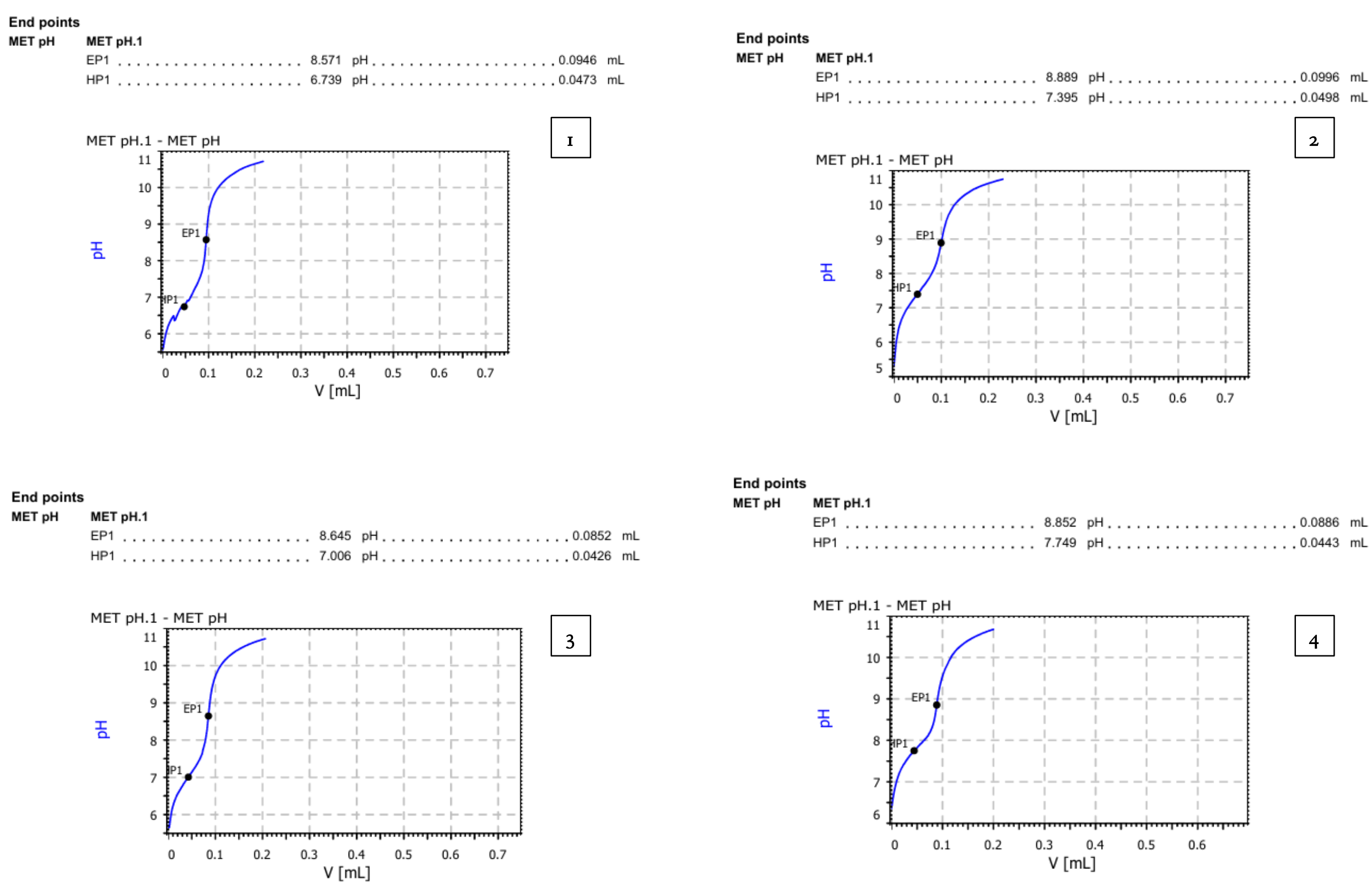

Figure S4. Potentiometric titrations of compounds **1-4** with standard NaOH 0.1 N. Experimental conditions: typically, in a 20-mL amber glass vial, the photoacid stock (5 mM) in MeOH (2.0 mL) is added to an aqueous solution of KCl (20 mM, 18.0 mL).

9. Molar Extinction coefficients of compounds 1-4

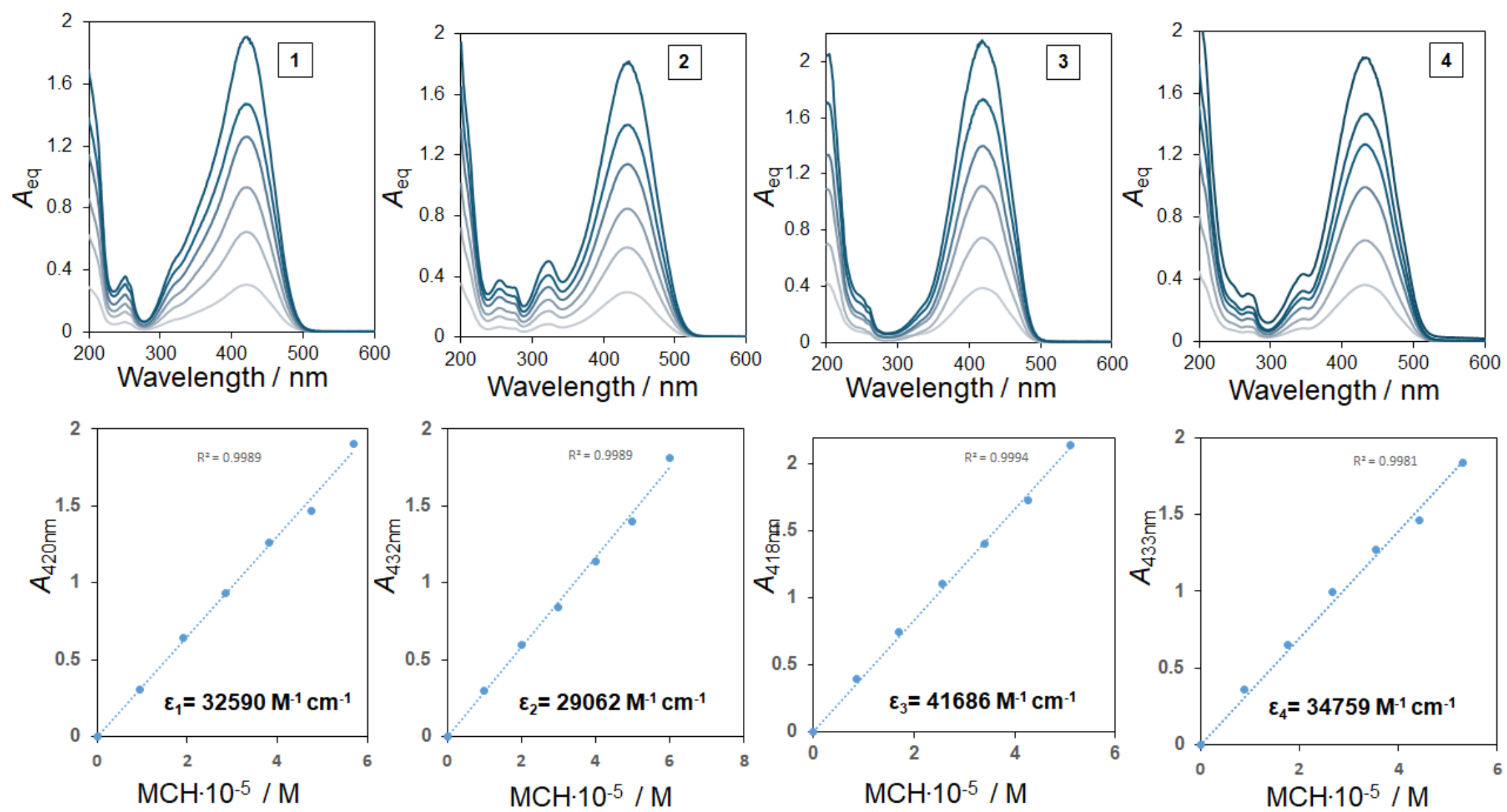

Figure S5. Representative spectra as a function of the concentration of **1-4** at equilibrium in the dark (top), and the corresponding calibration curve (bottom). Experimental conditions: [phosphate buffer] = 20 mM, pH = 4, T = 25 °C.

10. Isomerization under Light Irradiation (Quantum Yields of compounds 1-4)

The photoisomerization quantum yields (Φ) of compounds **1-4** were determined by monitoring the conversion of the protonated merocyanine (MCH) form to the spiropyran (SP) form under continuous irradiation at λ = 425 nm at pH 4 using UV-Vis absorption spectroscopy (Figure S7).

For the determination of Φ, irradiation was carried out under monochromatic and collimated light conditions, employing the experimental setup illustrated in Figure S10. In the present work, the light irradiation was provided by a 425 nm Prizmatix LED source equipped with a collimator and an iris diaphragm to obtain a well-defined and spatially confined excitation beam. The sample was irradiated in a 10 mm cuvette placed above the UV-Vis spectrometer sample compartment. The incident optical power (P_0) at the sample position was 100 μW, corresponding to the total absorption regime.

The photoisomerization quantum yield was calculated according to the following equation:

$$\square = -\frac{dA_{MCH}}{dt}\frac{h\,C\,V\,N_A}{\square_{MCH}\;d\;\square_{ex}\;P_0(1-10^{-A_{ex}})}$$

where Φ represents the quantum yield of the isomerization reaction, dA_{MCH}/dt and $\square_{MCH}$ are the rate of absorbance depletion and the molar absorptivity of the MCH form, and h, C, V, N_A, P_0 and d are the Planck constant, the concentration, the volume of the sample, the Avogadro number, the power of the LED light source and the optical path length of light through the sample, respectively. $\square_{ex}$ is the excitation wavelength, while A_{ex} is the absorbance of the photochemical system at $\square_{ex}$.

To ensure homogeneous mixing, the cuvette was fitted with an electronic cell stirrer and positioned within the spectrometer chamber, which was additionally equipped with a magnetic stirrer. Continuous

stirring was maintained throughout the irradiation period, providing homogeneous mixing and consistent exposure of the sample to the excitation light.

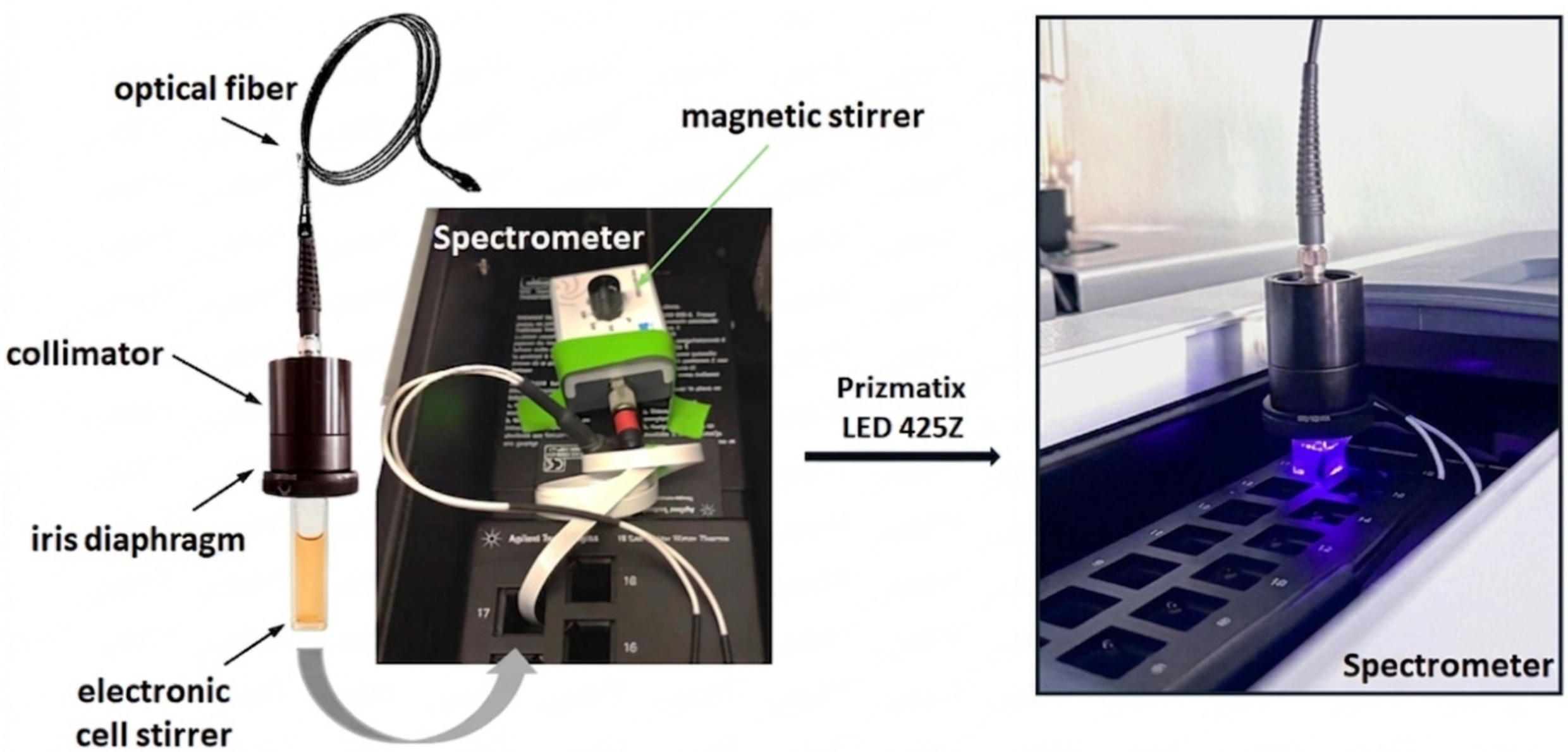

Figure S6. Experimental setup used for determination of photoisomerization quantum yields. A 425 nm LED light source coupled with a fiber-optic probe was positioned directly above the cuvette holder of the UV-Vis spectrometer.

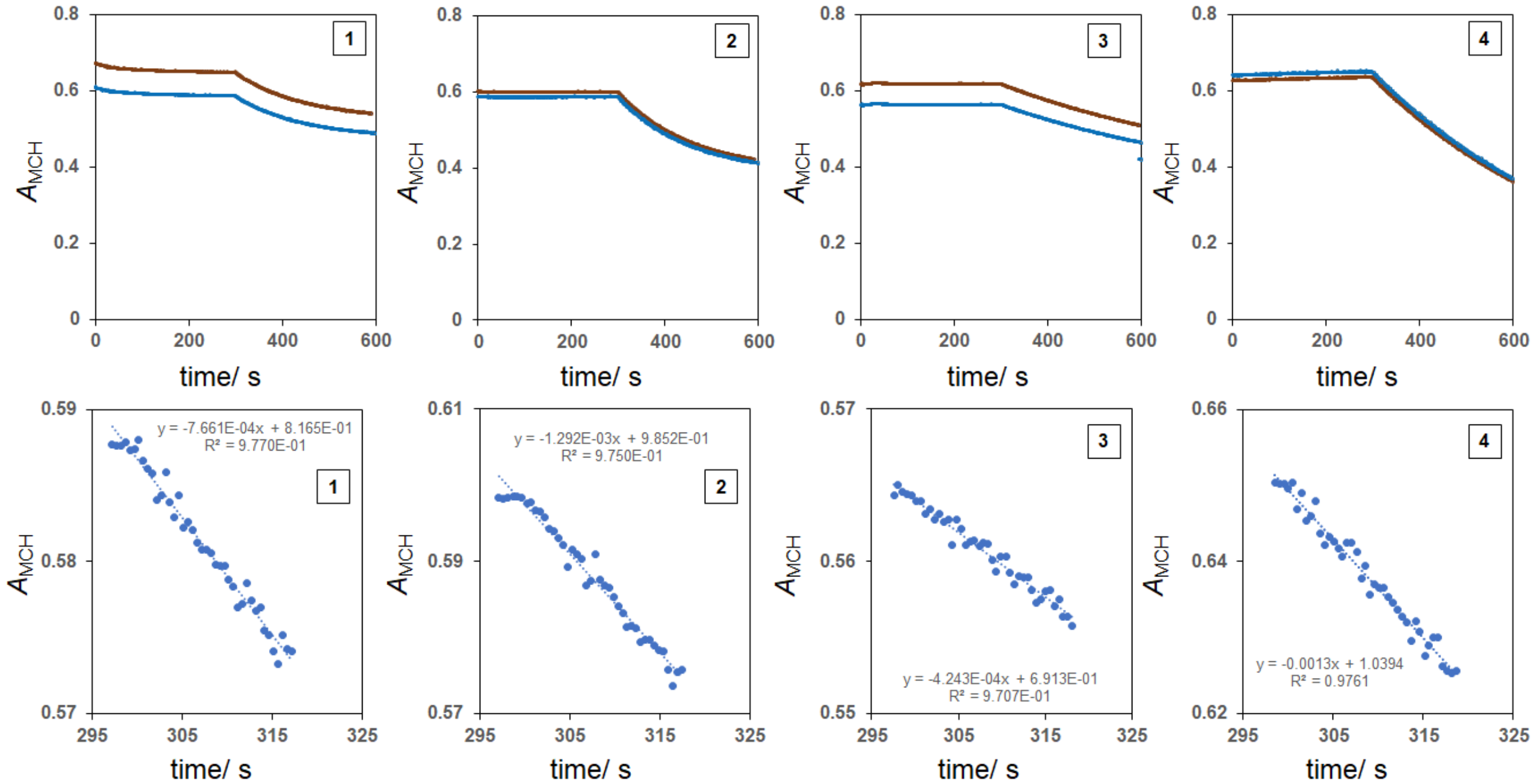

Figure S7. Absorbance profiles (at $\lambda_{max,MCH}$ and λ_{ex} = 425 nm) of compounds **1**-**4** over time under 425 LED light irradiation (top) and corresponding linear regime (bottom). The slope of the linear fit (dA_{MCH}/dt) is used in equation 4 to compute the quantum yield. Experimental conditions: [phosphate buffers] = 20 mM, pH = 4, T = 25 °C.

11. SP-to-MC(H) relaxation in the dark for compounds 1-4

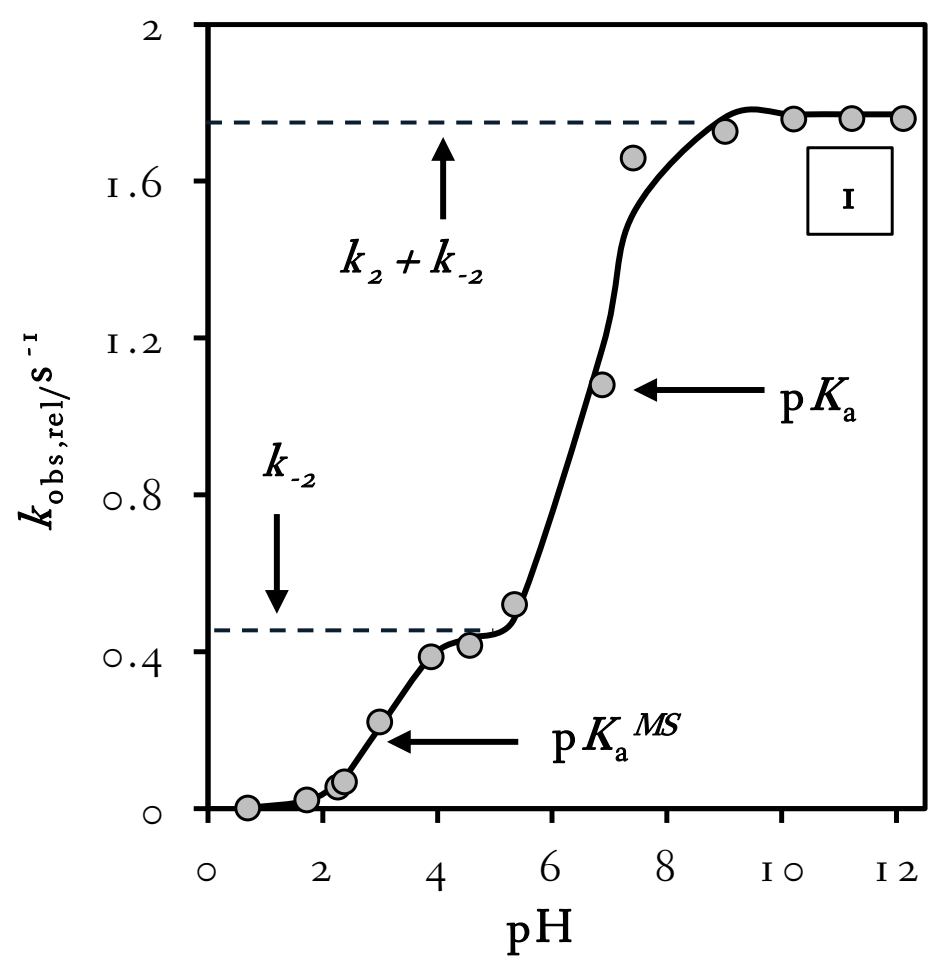

Fitted Values
pK_a^{MS} = 2.80 (2.9*)
pK_a = 6.78
K_c = 3.81 (3.80*)
k_{-2} = 0.44 (0.34*)

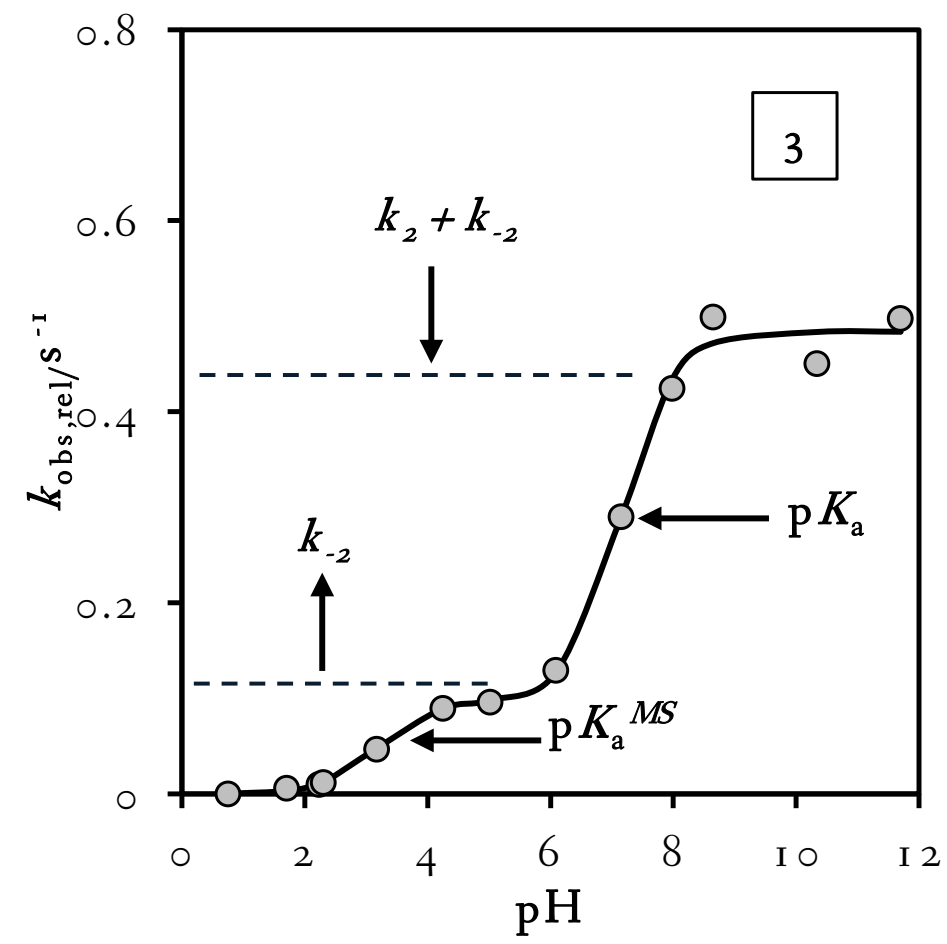

Fitted Values
pK_a^{MS} = 3.19 (3.2*)
pK_a = 7.15
K_c = 4 (3.7*)
k_{-2} = 0.096 (0.09*)

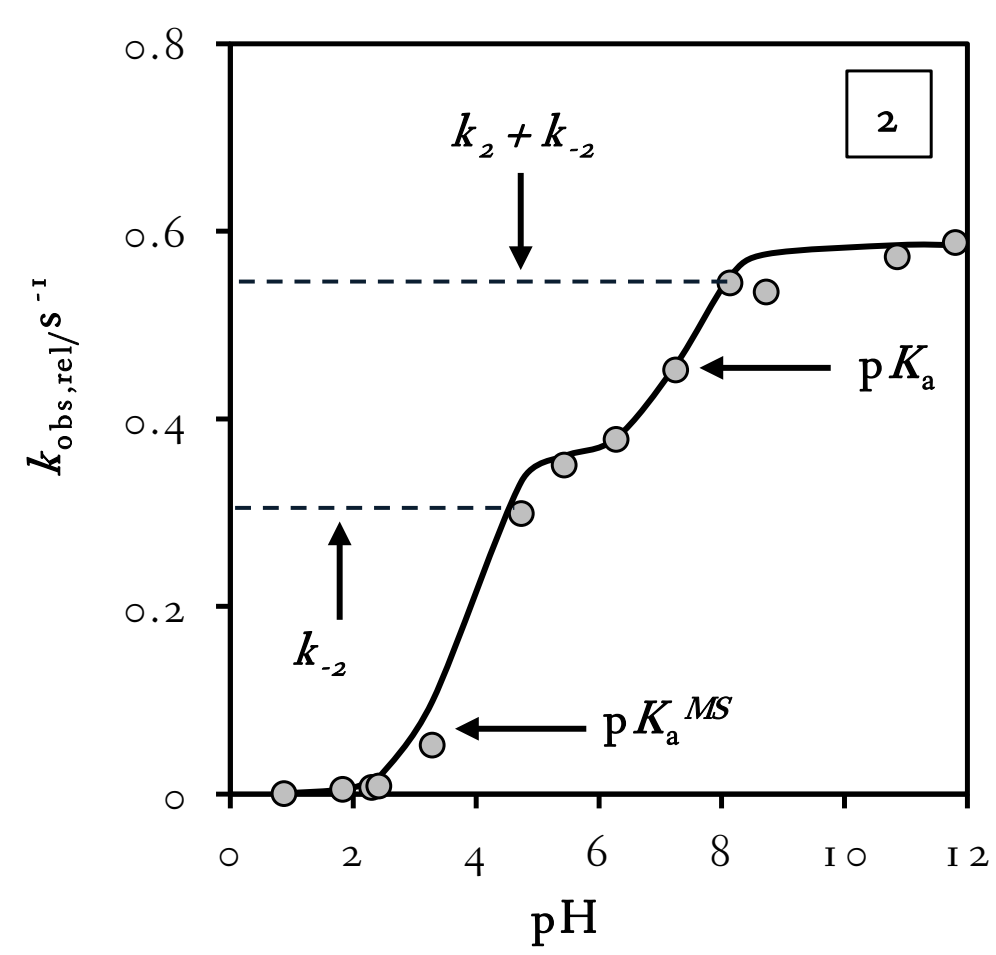

Fitted Values
pK_a^{MS} = 4 (4.0*)
pK_a = 7.39
K_c = 0.60 (0.6*)
k_{-2} = 0.36 (0.365*)

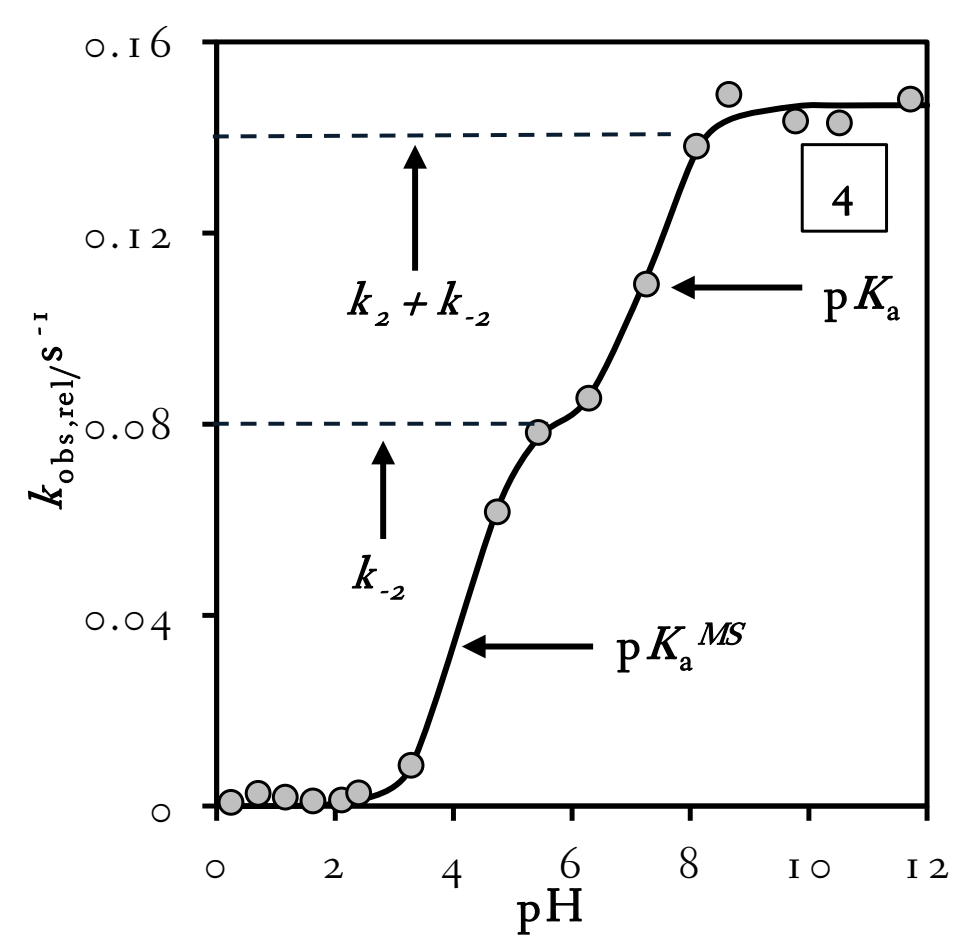

Fitted Values
pK_a^{MS} = 4.22 (3.9*)
pK_a = 7.33
K_c = 0.80 (0.7*)
k_{-2} = 0.080 (0.083*)

Figure S8. Relaxation profiles of 1-4 as a function of the pH after light irradiation; the solid black line represents the best fit to equation 5. *Indicates the values independently obtained from UV-Vis

experiments (see Figures S1-S3). The fitted values are in good agreement with these independently measured data. Experimental conditions: [**1**-**4**] = 25 ± 2 μM, [phosphate buffers] = 20 mM (2 < pH < 10) *T* = 25 °C.

12. Hydrolysis of 1-4

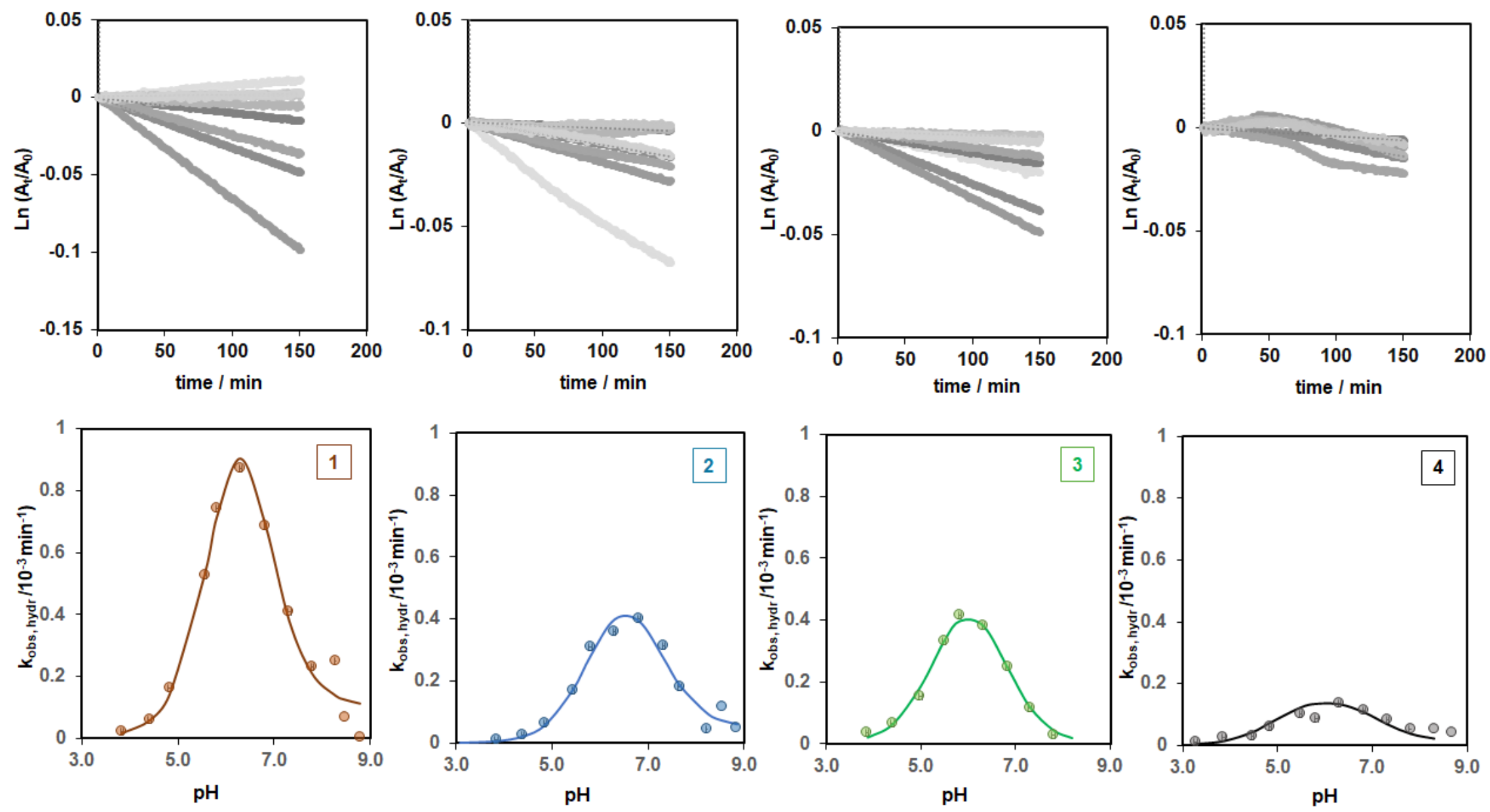

Figure S9. Linearized kinetic profiles for the hydrolysis of **1**, **2**, **3** and **4** at different pH in the dark (top). Plots of the obtained apparent first-order rate constant of hydrolysis as a function of the pH and relative fitting (bottom); solid-colored lines represent the best fit to equation 6. Experimental conditions: [**1**-**4**] = 25 ± 2 μM, [phosphate buffers] = 20 mM (3 < pH < 9), T = 25 °C; data collected the maximum of the corresponding MCH (3 < pH < 9) and MC form (pH > 7).

13. Solubility and pH Jump Setup

The solubility (S_{MCH}) of **4** in its MCH form was determined by suspending an excess amount of crystals in 1 mL of 20 mM KCl in 40% MeOH:H_2O (+1 μL of freshly made HCl 1 M). The suspension was allowed to equilibrate until the pH stabilized. The solution was then filtered through a 0.22 μm PTFE filter and analyzed by UV-Vis spectroscopy after dilution. Based on the measurements shown in Figure 10a, the solubility of the MCH form was determined to be S_{MCH} = 1.02 ± 0.01 mM.

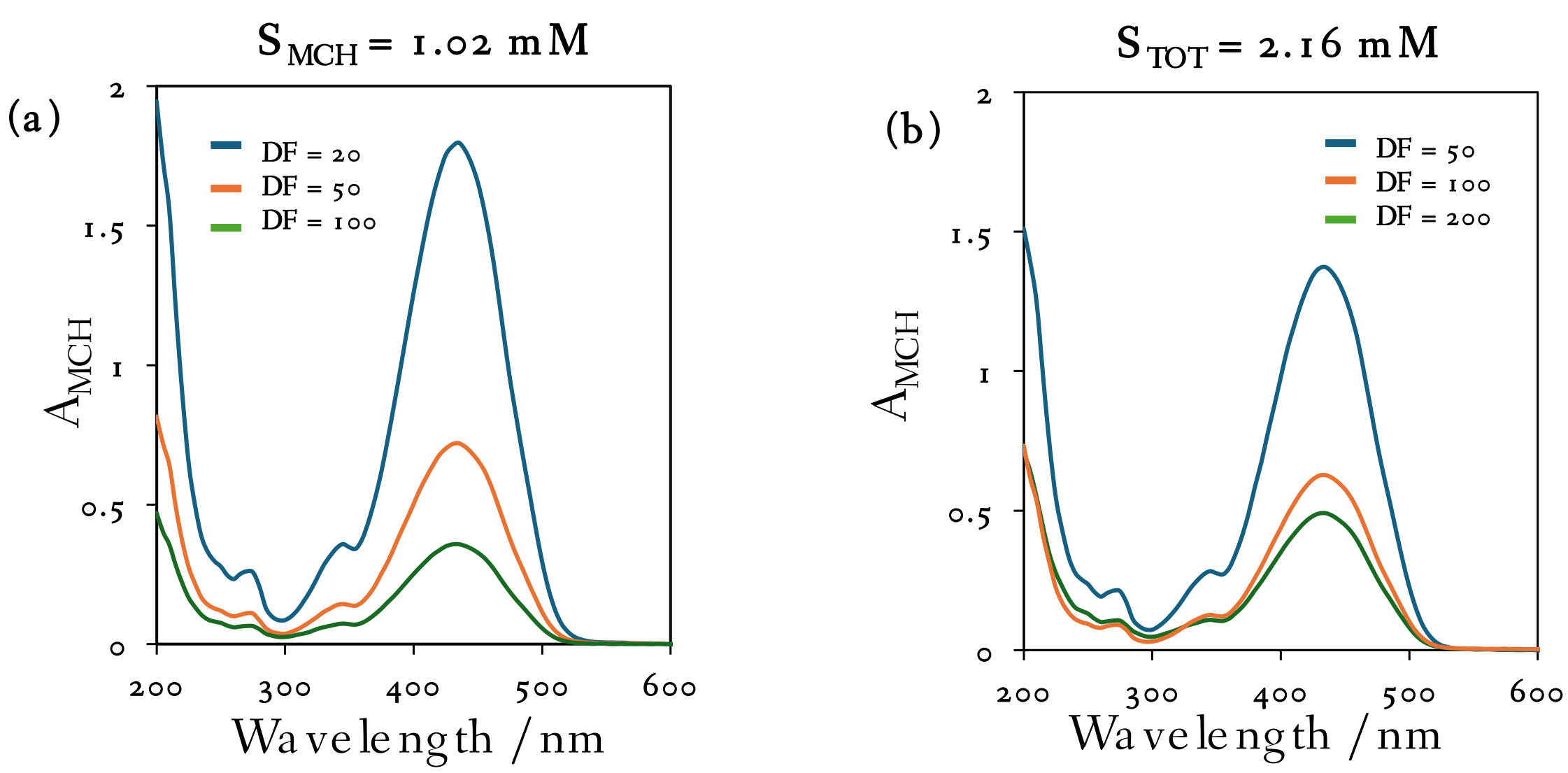

$\square_{MCH_4}$ $(L\ mol^{-1}\ cm^{-1}) = 34759$

Figure S10: UV-Vis spectra were recorded after filtration, and the solubility was calculated using three independent (triplicate) experiments with dilution factors (DF). Experimental conditions: [phosphate buffer] = 20 mM, pH = 4.5, T = 25 °C.

The experiment was subsequently repeated in the presence of 1 equivalent of KOH to reach pH ≈ pK_a^{GS}. For this purpose, KOH (10.2 μL, 0.1 N) was added to 1 mL of 20 mM KCl in 40% MeOH/H_2O solution. After pH equilibration, the solution was filtered and UV-Vis absorption spectra were recorded again after dilution. The calculated total solubility obtained from these measurements was S_{TOT} = 2.2 ± 0.4 mM, as illustrated in Figure 10b. This value is consistent with the expected value (≈ 2 × S_{MCH} ≈ 2.04 mM), confirming that S_{MCH} is approximately 1 mM under these conditions.[12]

Following the solubility measurements, the solution was subjected to a pH-jump experiment, transferring the vial into a 50-mL jacketed beaker kept at 25 °C, equipped with a 2.5 cm cross-shaped stir bar and mounted above a 45° mirror cage. pH data acquisition was carried out under stirring (450 rpm) and inert atmosphere (gentle Ar bubbling) with a pre-calibrated Unitrode Pt1000 glass electrode (Figure S11). The pH changed from pH_{dark} = 7.6 to pH_{light} = 4.6, which was used as a reference for the photochemical switching behavior.

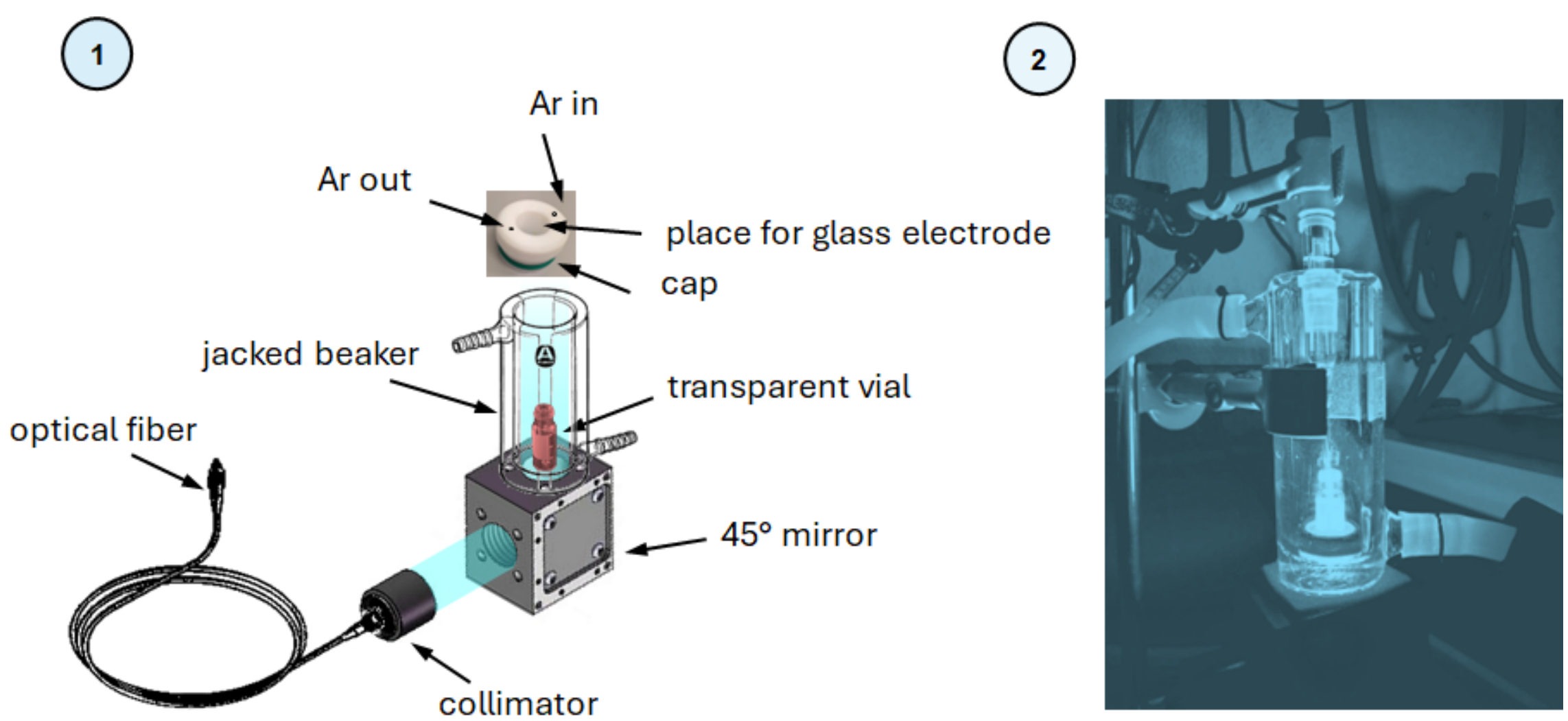

Figure S11: The apparatus used for the pH jump experiments consists of an (1) optical fiber equipped with a collimator that directs light into a 45° mirror cage, ensuring accurate alignment of the light beam through the vial. (2) The vial is maintained at a constant temperature of 25 °C using a thermostat jacketed beaker. To minimize atmospheric interference, the system is kept under an argon atmosphere using a custom-made gas-tight cap designed to accommodate the pH electrode.

14. Computational Data

Table S3: Free energies are reported in kcal mol^{-1} and are defined as $\Delta G_{MC \to SP} = G_{SP} - G_{MC}$; therefore, negative values indicate preferential stabilization of the closed SP form. Experimental values were calculated at 298.15 K using $\Delta G_{exp} = -RT \ln K_c$, using the independently determined K_c values reported in the manuscript. The compound numbering and R^1/R^2 substitution patterns follow Scheme S1.

Compound	$K_{c,exp}$	ΔG_{exp}	ΔG_{calc} Gas phase	ΔG_{calc} SMD
1HH	3.80	−0.79	−13.00	−2.47
2OMeH	0.60	+0.30	−12.04	−1.47
3HOMe	3.70	−0.78	−11.22	−2.07
4OMeOMe	0.70	+0.21	−10.25	−0.82

Table S4. Local C–O bond metrics for the methoxy substituents at positions R^1 and R^2 in the merocyanine (MC) and spiropyran (SP) forms of compound **4**, calculated in water using the SMD solvation model. Reported values include the aryl–O bond length, Wiberg bond index, and occupancy of the p-type oxygen lone pair, LP(2), involved in π donation. The MC–SP differences indicate a larger change in conjugative engagement for the R^1 methoxy group than for the R^2 substituent.

Descriptor	R^1-OMe MC	R^1-OMe SP	Change	R^2-OMe MC	R^2-OMe SP	Change
Aryl–O distance / Å	1.3668	1.3772	−0.0104	1.3604	1.3622	−0.0018
Aryl–O Wiberg index	1.0035	0.9798	+0.0237	1.0159	1.0102	+0.0057
LP(2) occupancy / e	1.85905	1.87338	−0.01433	1.84573	1.85340	−0.00767

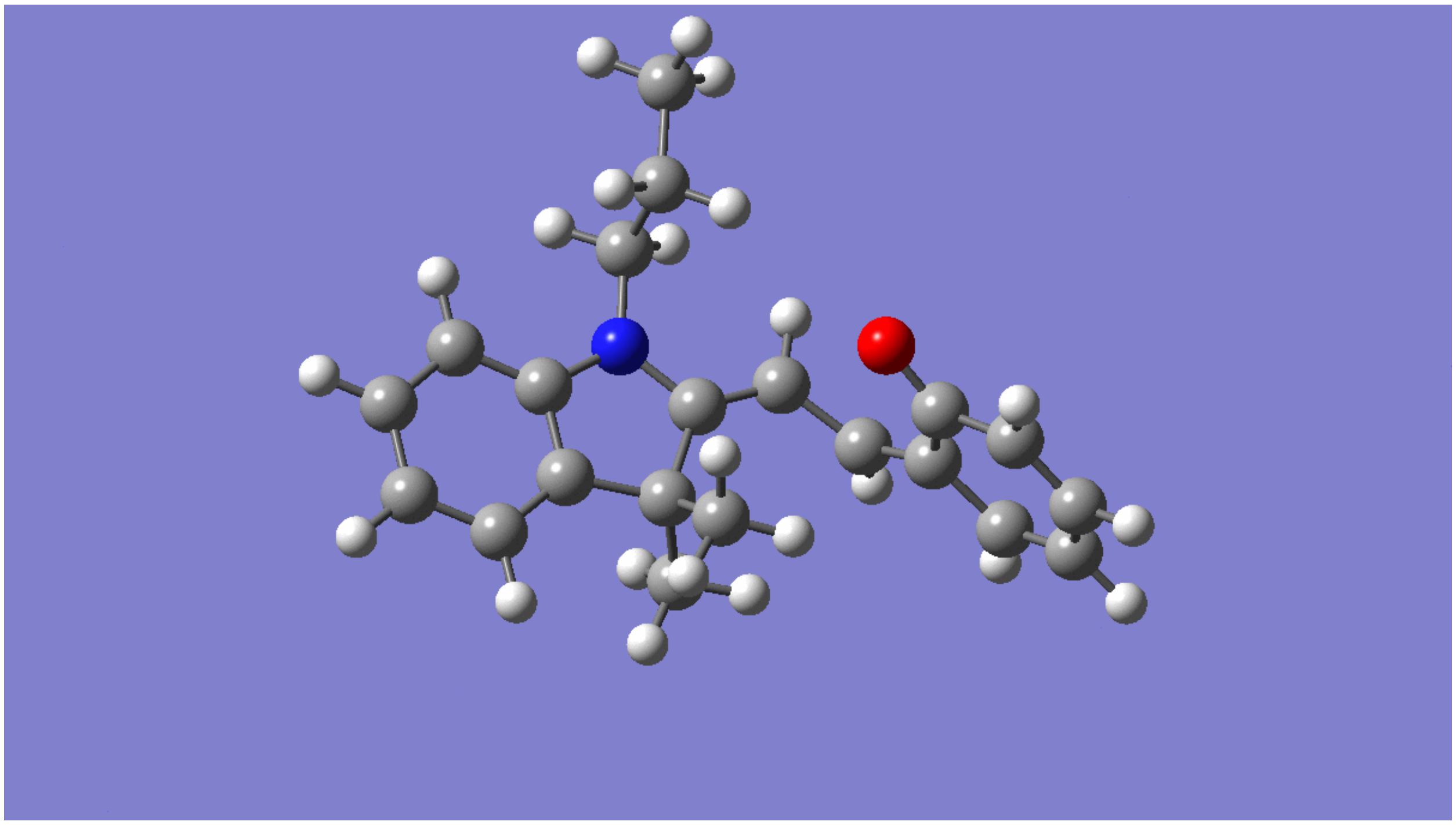

Figure S12. Transition States of compounds 1-4.

Table S5. Calculated free-energy barriers and vibrational characterization of TS for compounds **1**-**4** at the B97X-D/6-31+G(d,p)/SMD (water) level. Free energy separations are reported in kcal/mol. Each transition state exhibits a single imaginary frequency. The free energy between TS and SP is only a formal energy difference and does not represent an elementary activation barrier. Optimization following displacement along the ring-closing direction leads directly to SP for compounds **1** and **3**, whereas high-energy cisoid minima are obtained for compounds **2** and **4**.

Compound	$\Delta G^{\ddagger}_{trans\rightarrow TS}$	***Formal*** $\Delta G^{\ddagger}_{SP\rightarrow TS}$	***Frequency (cm^{-1})***	***Optimization following the closure-side displacement***
1	***20.45***	*22.92*	*−186.7*	*SP*
2	***21.03***	*22.50*	*−207.0*	*Cisoid minimum*
3	***18.79***	*20.86*	*−202.0*	*SP*
4	***19.90***	*20.72*	*−221.2*	*Cisoid minimum*

Table S6. Effect of methoxy substitution at R[1] on the calculated *trans*-merocyanine-to-TS activation free energy. The activation energy difference is defined for each R[2] fixed substituent and is reported in kcal/mol. The corresponding predicted rate ratio was estimated at 298.15 K using the Eyring equation, assuming identical prefactors and transmission coefficients.

Fixed R²	***Matched comparison***	$\Delta\Delta G^{\ddagger}_{trans \rightarrow TS}$	$k_{2,R1=H}/k_{2,R1=OMe}$
H	***1 (HH) → 2 (OMeH)***	***+0.58***	***2.7***
OMe	***3 (HOMe) → 4 (OMeOMe)***	***+1.12***	***6.6***

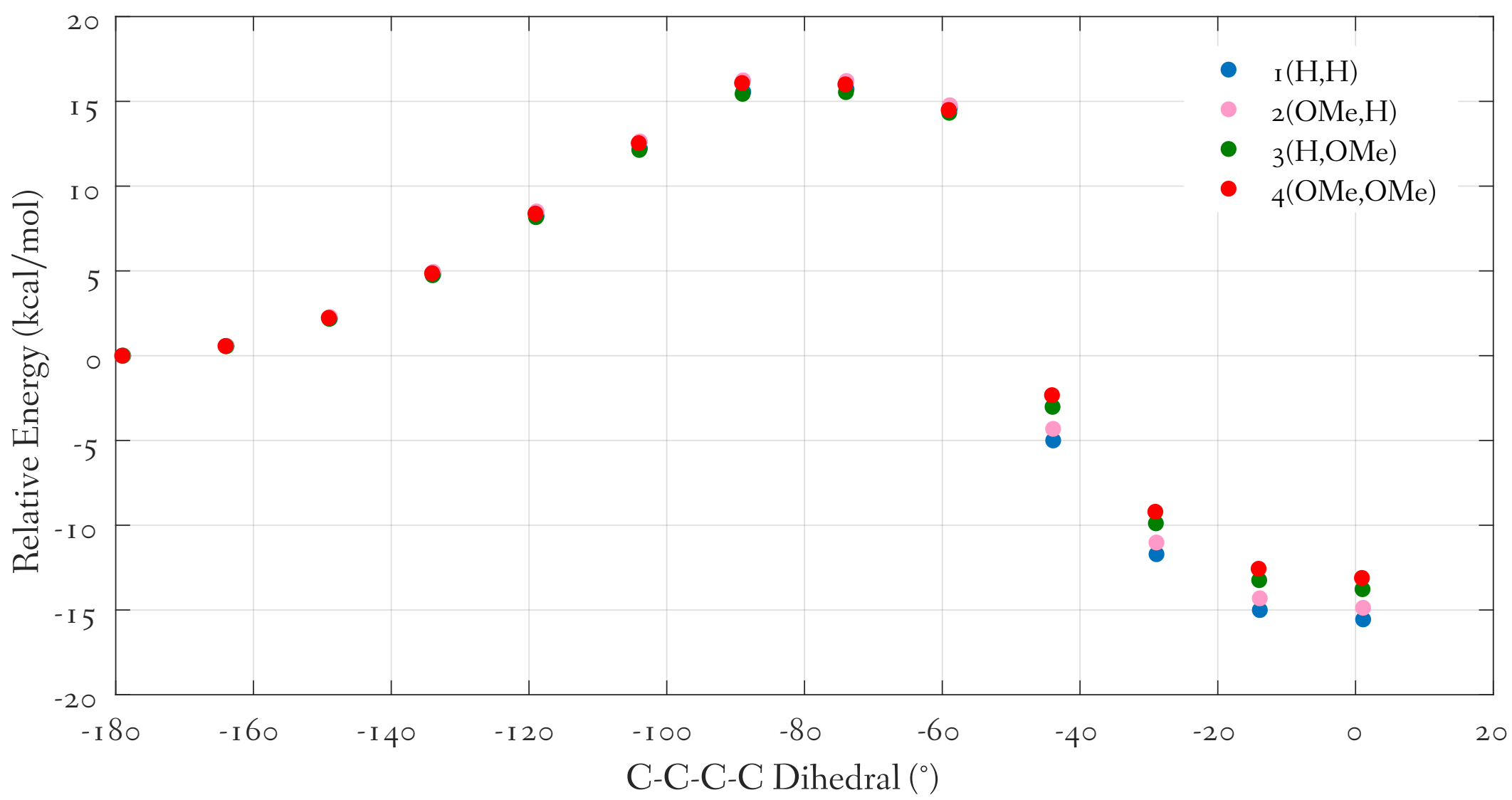

Figure S13. Relaxed torsional energy profiles for compounds **1-4** as a function of the C–C–C–C dihedral angle connecting the transoid and cisoid configurations of the merocyanine. For each compound, the electronic energies were referenced to the corresponding trans-merocyanine minimum at -180°. The profiles show a broad maximum of approximately 15-16 kcal mol in the region to -90°, -60°. The abrupt decrease observed upon further rotation reflects relaxation away from the open-merocyanine scan branch toward the lower-energy ring-closed basin. Compound assignments are **1** (H,H), **2** (OMe,H), **3** (H,OMe), and **4** (OMe,OMe).

15. ^{1}H and ^{13}C NMR spectra

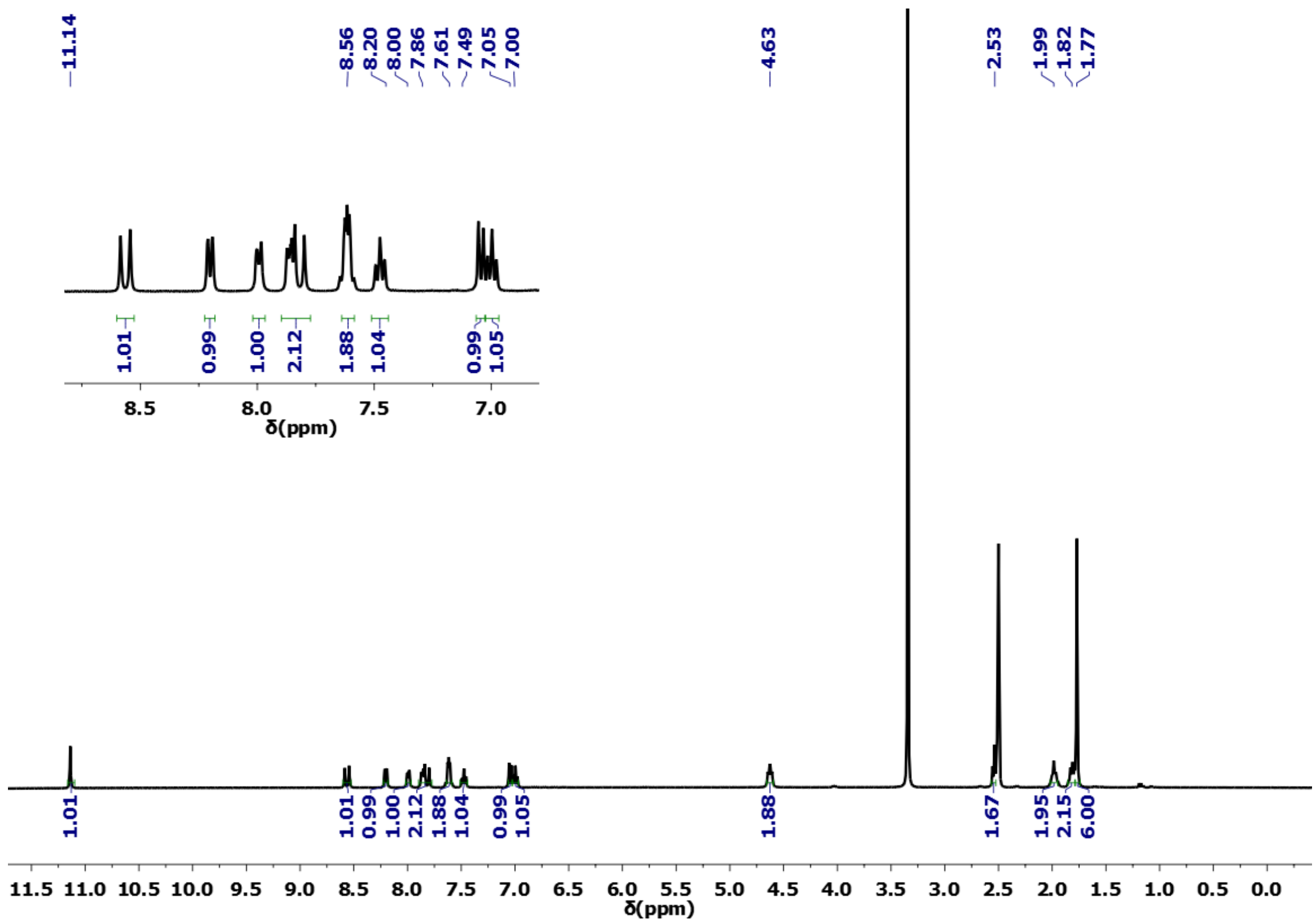

Figure S14. ^{1}H NMR spectrum (400 MHz, DMSO-d_6) of compound **1**.

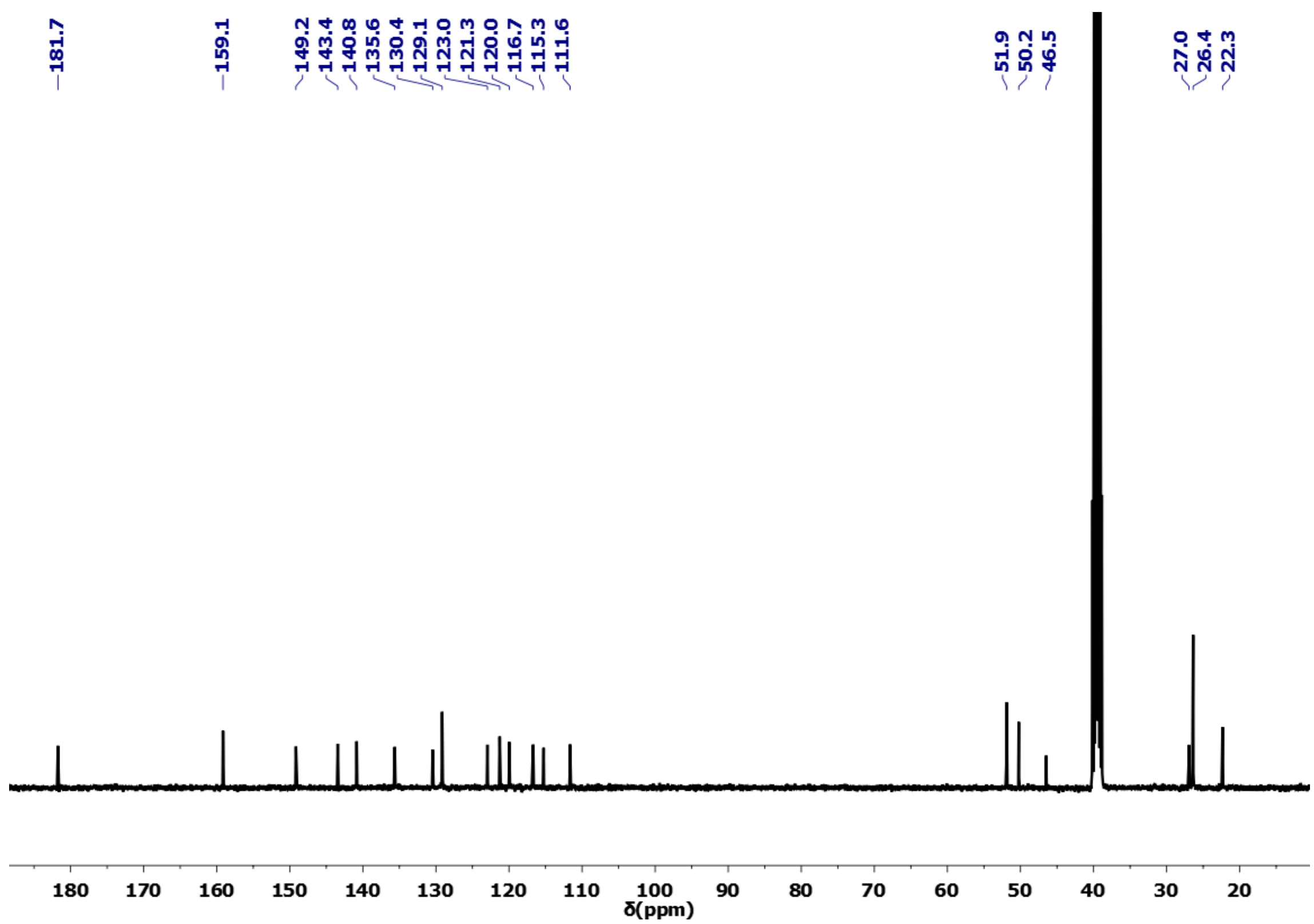

Figure S15. ^{13}C NMR spectrum (101 MHz, DMSO-d_6) of compound **1**.

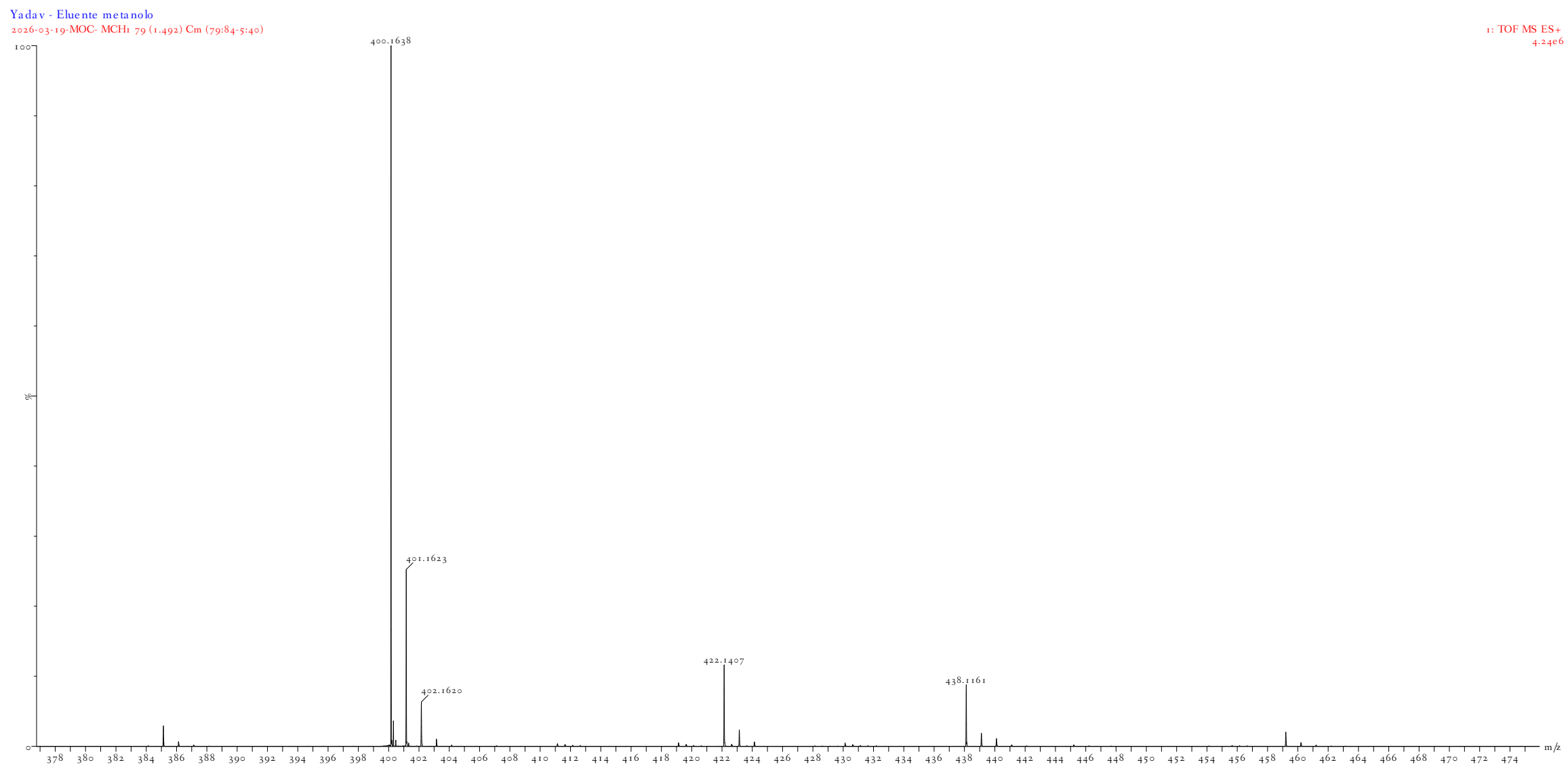

Figure S16. HRMS (TOF-MS, ES$^+$) spectrum of compound **1**.

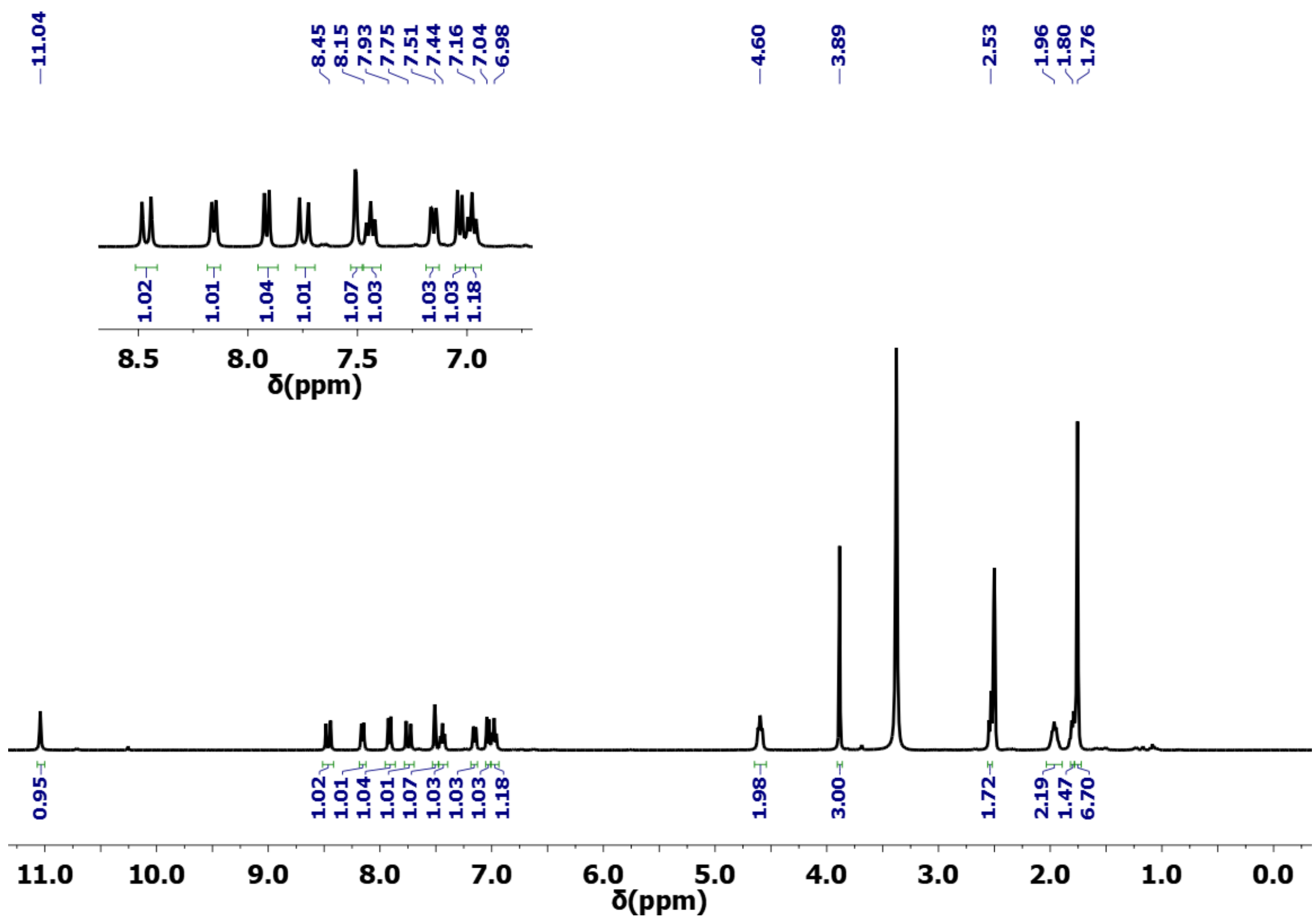

Figure S17. ^{1}H NMR spectrum (400 MHz, DMSO-d_6) of compound **2**.

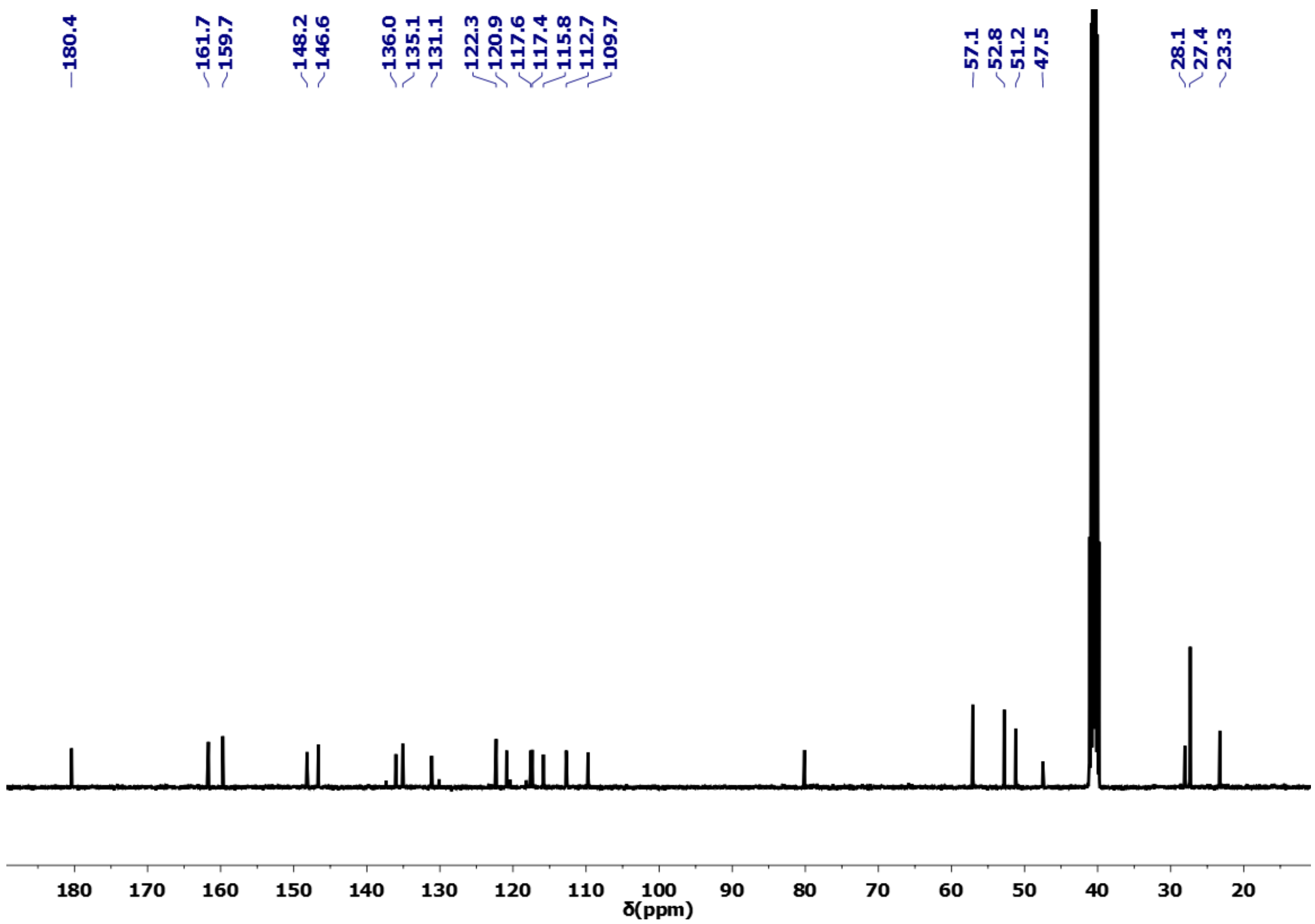

Figure S18. ^{13}C NMR spectrum (101 MHz, DMSO-d_6) of compound **2**.

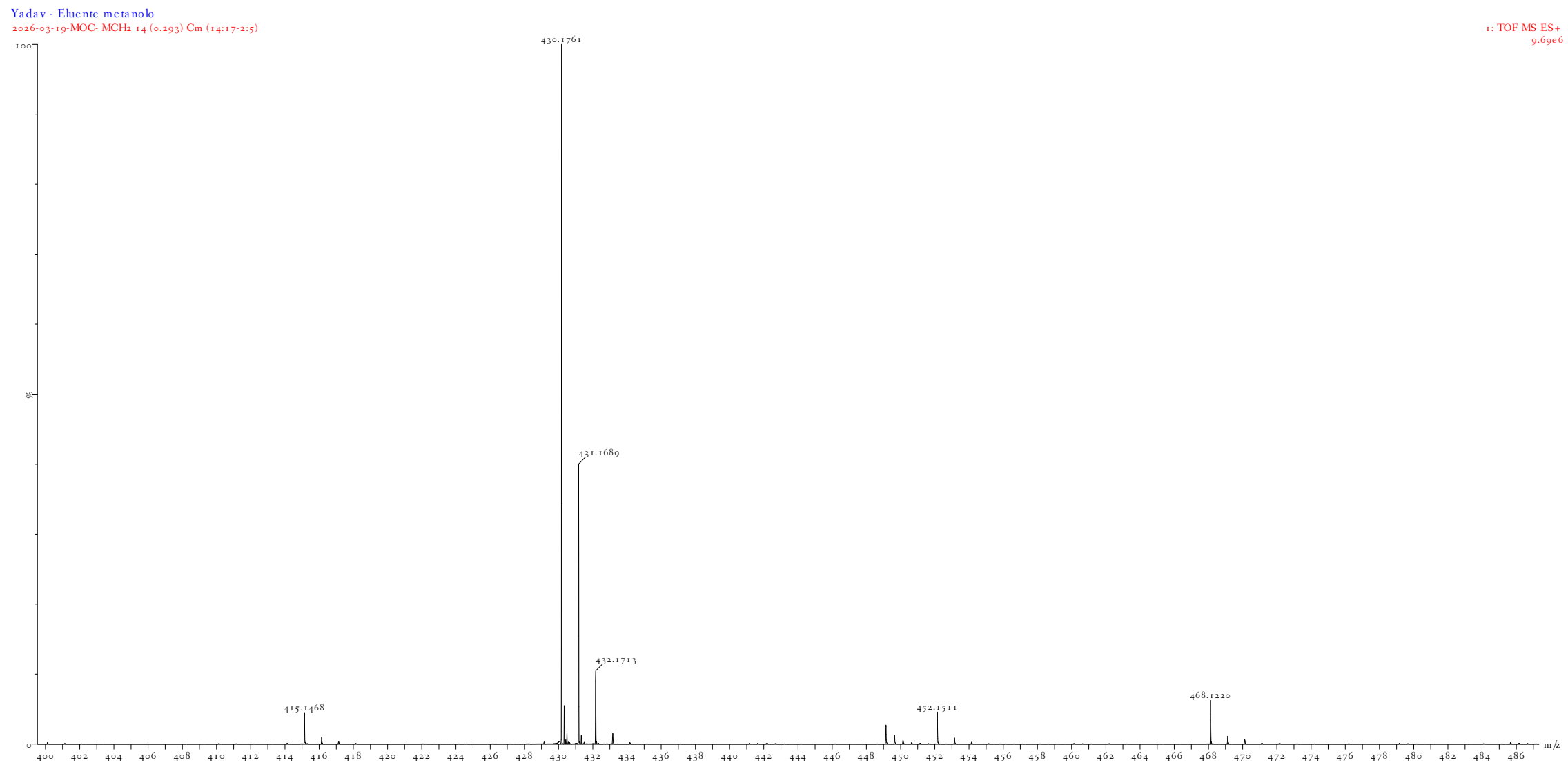

Figure S19. HRMS (TOF-MS, ES$^+$) spectrum of compound **2**.

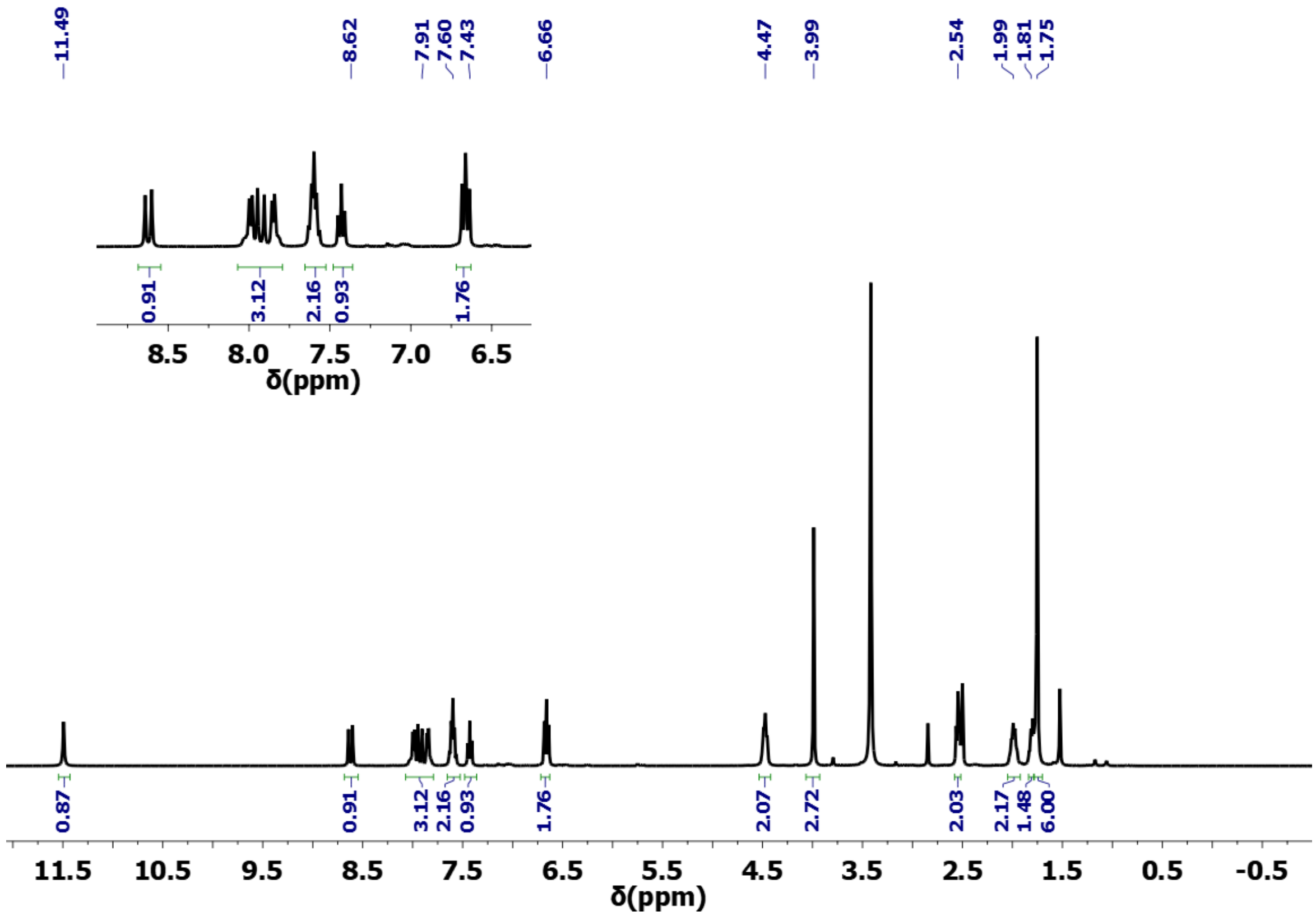

Figure S20. ^{1}H NMR spectrum (400 MHz, DMSO-d_6) of compound **3**.

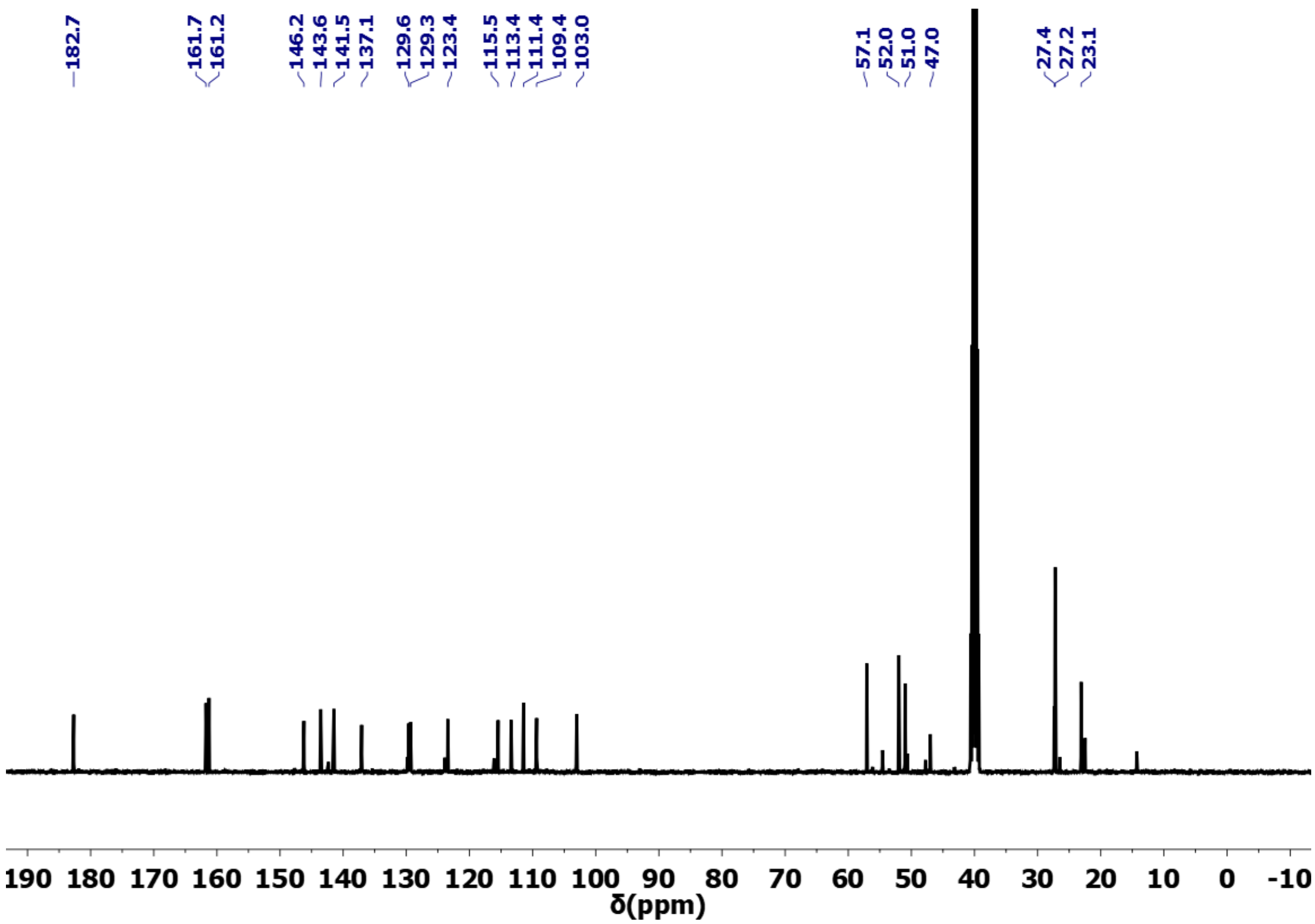

Figure S21. ^{13}C NMR spectrum (101 MHz, DMSO-d_6) of compound **3**.

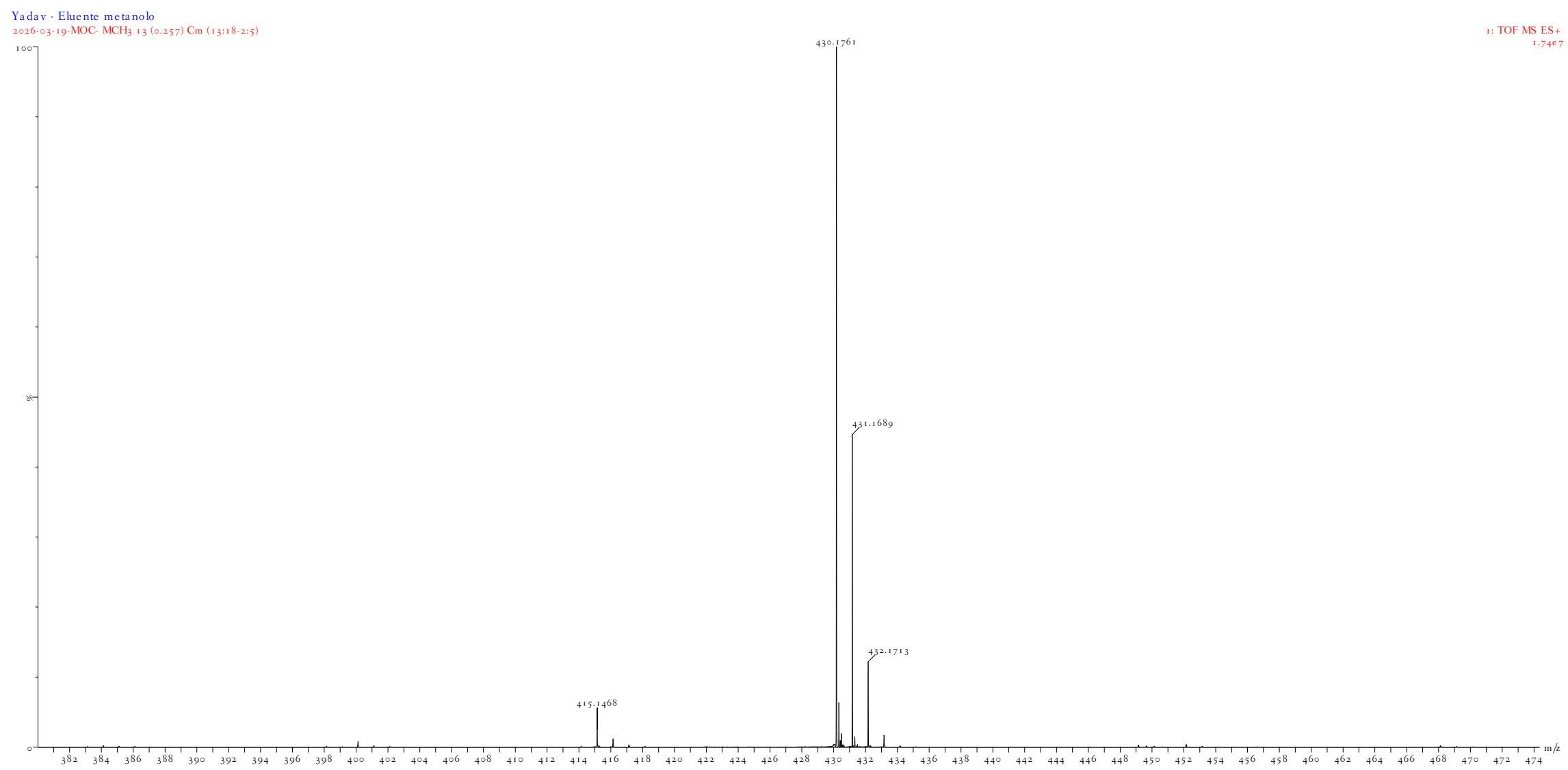

Figure S22. HRMS (TOF-MS, ES$^+$) spectrum of compound **3**.

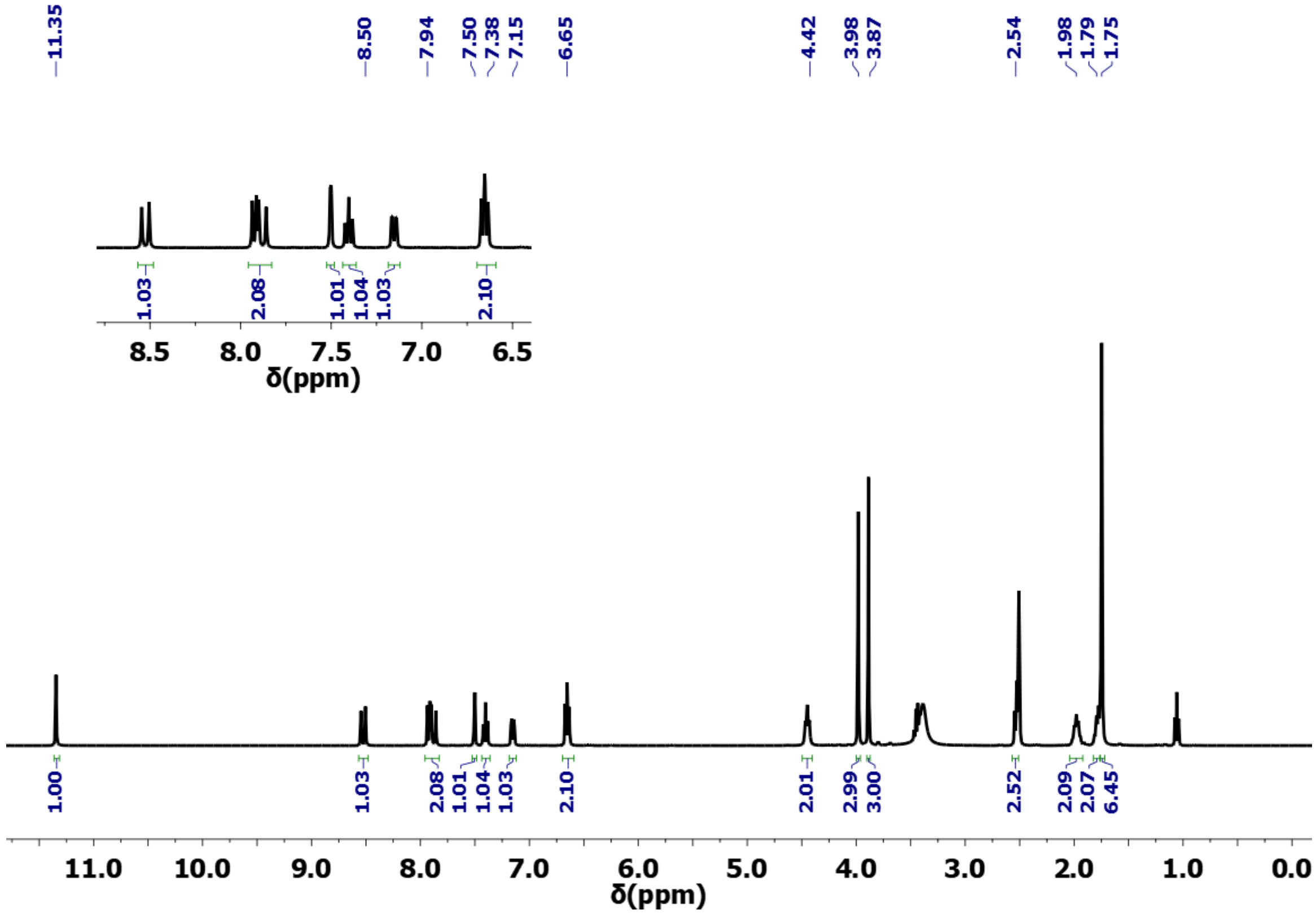

Figure S23. ^{1}H NMR spectrum (400 MHz, DMSO-d_6) of compound **4**.

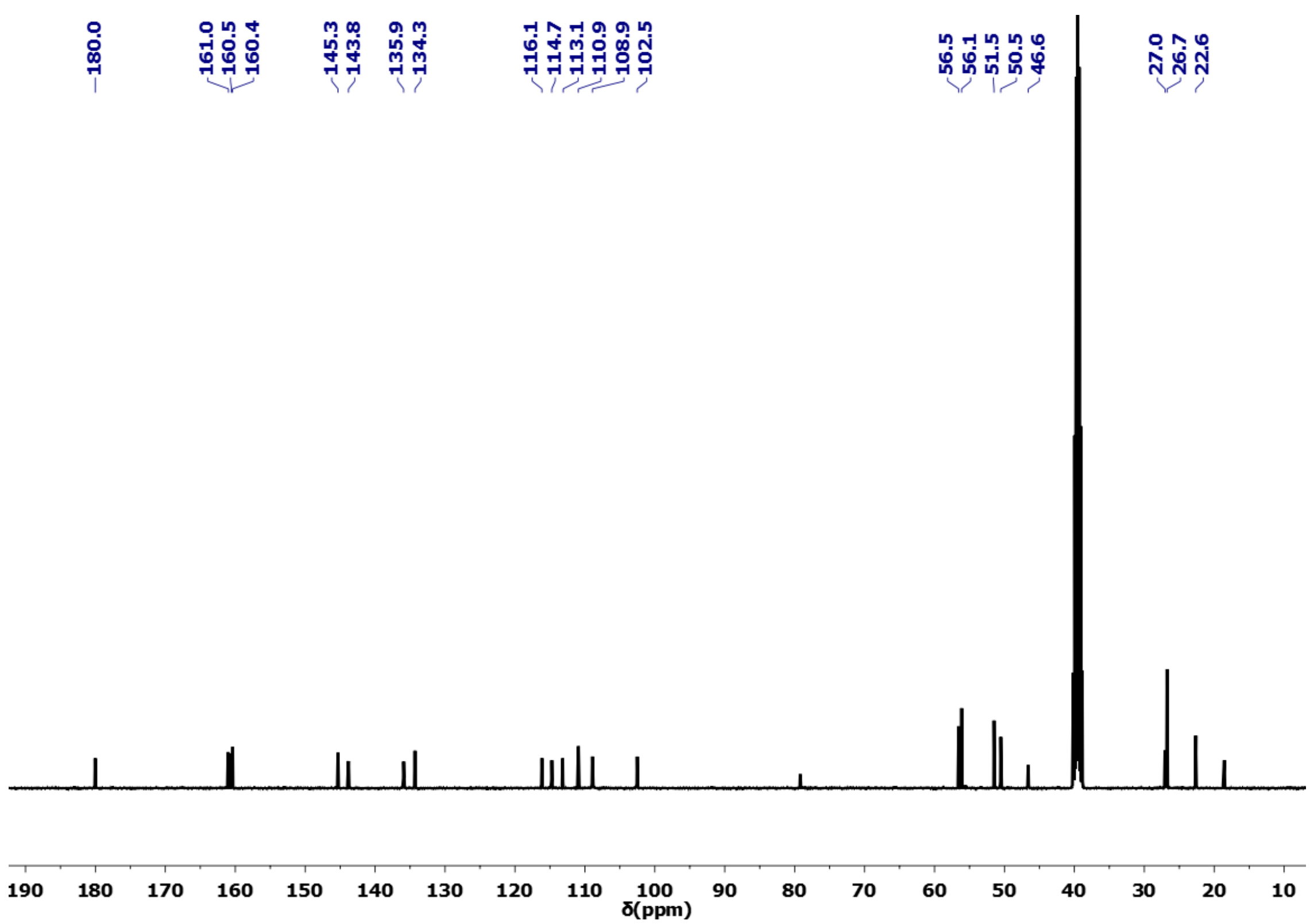

Figure S24. ^{13}C NMR spectrum (101 MHz, DMSO-d_6) of compound **4**.

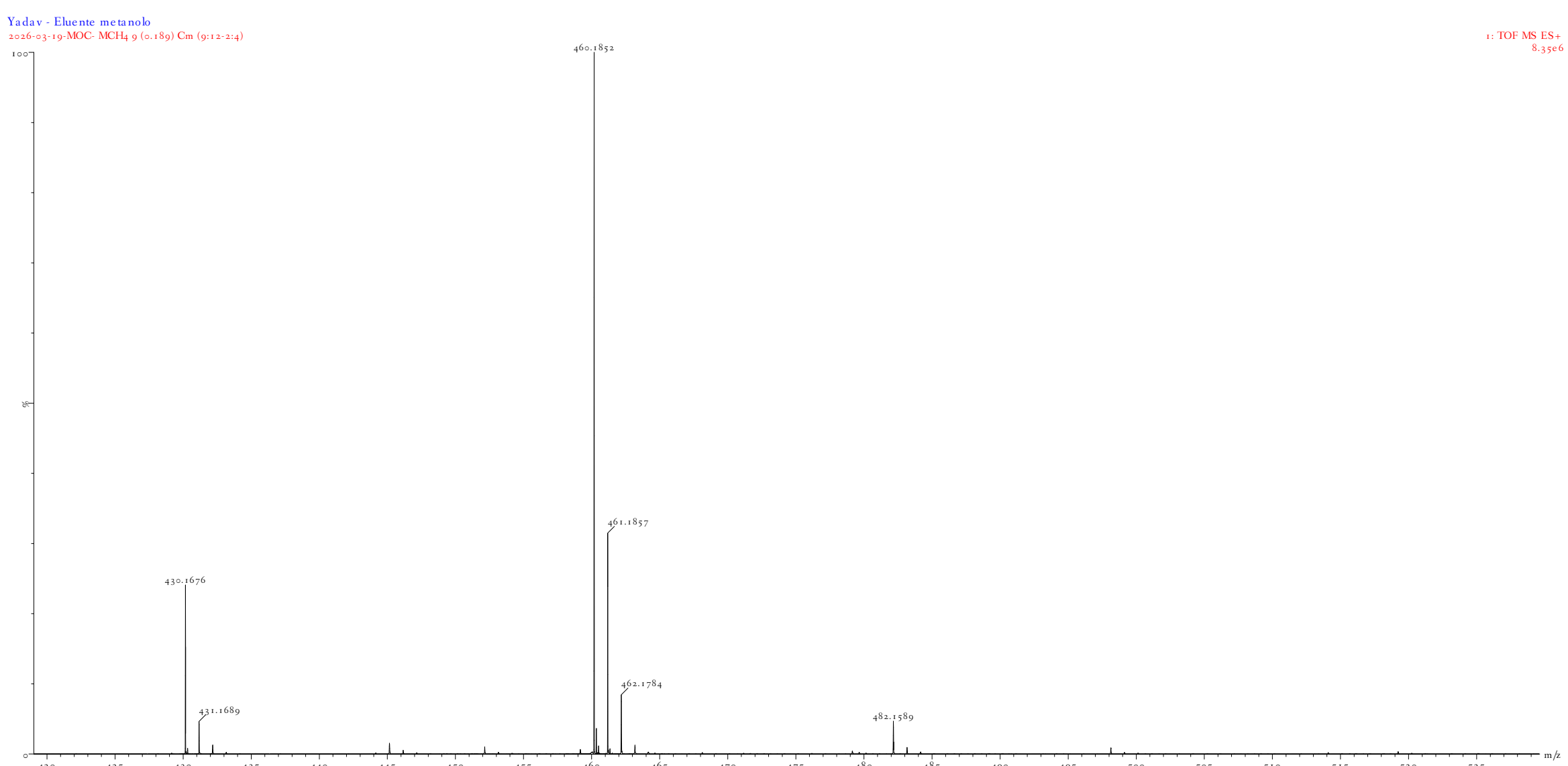

Figure S25. HRMS (TOF-MS, ES^+) spectrum of compound **4**.

16. References

1) E. I. Balmond, B. K. Tautges, A. L. Faulkner, V. W. Or, B. M. Hodur, J. T. Shaw and A. Y. Louie, *J. Org. Chem.*, 2016, **81**, 8744-8758.
2) L. Wimberger, S. K. K. Prasad, M. D. Peeks, J. Andréasson, T. W. Schmidt and J. E. Beves, *J. Am. Chem. Soc.*, 2021, **143**, 20758-20768.
3) C. Berton, D. M. Busiello, S. Zamuner, E. Solari, R. Scopelliti, F. Fadaei-Tirani, K. Severin and C. Pezzato, *Chem. Sci.*, 2020, **11**, 8457-8468.
4) Gaussian 16, Revision B.01, M. J. Frisch, G. W. Trucks, H. B. Schlegel, G. E. Scuseria, M. A. Robb, J. R. Cheeseman, G. Scalmani, V. Barone, G. A. Petersson, H. Nakatsuji, X. Li, M. Caricato, A. V. Marenich, J. Bloino, B. G. Janesko, R. Gomperts, B. Mennucci, H. P. Hratchian, J. V. Ortiz, A. F. Izmaylov, J. L. Sonnenberg, D. Williams-Young, F. Ding, F.

Lipparini, F. Egidi, J. Goings, B. Peng, A. Petrone, T. Henderson, D. Ranasinghe, V. G. Zakrzewski, J. Gao, N. Rega, G. Zheng, W. Liang, M. Hada, M. Ehara, K. Toyota, R. Fukuda, J. Hasegawa, M. Ishida, T. Nakajima, Y. Honda, O. Kitao, H. Nakai, T. Vreven, K. Throssell, J. A. Montgomery, Jr., J. E. Peralta, F. Ogliaro, M. J. Bearpark, J. J. Heyd, E. N. Brothers, K. N. Kudin, V. N. Staroverov, T. A. Keith, R. Kobayashi, J. Normand, K. Raghavachari, A. P. Rendell, J. C. Burant, S. S. Iyengar, J. Tomasi, M. Cossi, J. M. Millam, M. Klene, C. Adamo, R. Cammi, J. W. Ochterski, R. L. Martin, K. Morokuma, O. Farkas, J. B. Foresman, and D. J. Fox, Gaussian, Inc., Wallingford CT, 2016.

5) A. V. Marenich, C. J. Cramer and D. G. Truhlar, *J. Phys. Chem. B*, 2009, **113**, 6378–6396.

6) NBO Version 3.1, E. D. Glendening, A. E. Reed, J. E. Carpenter and F. Weinhold.

7) A. E. Reed, L. A. Curtiss, and F. Weinhold, *Chem. Rev.*, 1988, **88,** 899-926.

8) L. Wimberger, J. Andréasson, and J. E. Beves, *Chem. Commun.*, 2022, **58**, 5610-5613.

9) O. V. Dolomanov, L. J. Bourhis, R. J. Gildea, J. A. K. Howard and H. Puschmann, *J. Appl. Cryst.*, 2009, **42**, 339–341.

10) G. M. Sheldrick, *Acta Cryst. A*, 2015, **71**, 3–8.

11) G. M. Sheldrick, *Acta Cryst C*, 2015, **71**, 3–8.

12) C. Berton, D. M. Busiello, S. Zamuner, R. Scopelliti, F. Fadaei-Tirani, K. Severin and C. Pezzato, *Angew. Chem. Int. Ed.*, 2021, **60**, 21737-21740.
